# Multi-Strategy Assessment of Different Uses of QSAR under REACH Analysis of Alternatives to Advance Information Transparency

**DOI:** 10.3390/ijerph19074338

**Published:** 2022-04-04

**Authors:** Kazue Chinen, Timothy Malloy

**Affiliations:** 1 Institute of the Environment and Sustainability, University of California, Los Angeles, CA 90024, USA; 2 School of Law, University of California Los Angeles, Los Angeles, CA 90095, USA; malloy@law.ucla.edu; 3 Fielding School of Public Health, University of California Los Angeles, Los Angeles, CA 90092, USA

**Keywords:** QSAR, analysis of alternatives, REACH, weight of evidence, integrating testing strategy, battery testing, QPRF

## Abstract

Under the Registration, Evaluation, Authorization, and Restriction of Chemicals (REACH) analysis of alternatives (AoA) process, quantitative structure–activity relationship (QSAR) models play an important role in expanding information gathering and organizing frameworks. Increasingly recognized as an alternative to testing under registration. QSARs have become a relevant tool in bridging data gaps and supporting weight of evidence (WoE) when assessing alternative substances. Additionally, QSARs are growing in importance in integrated testing strategies (ITS). For example, the REACH ITS framework for specific endpoints directs registrants to consider non-testing results, including QSAR predictions, when deciding if further animal testing is needed. Despite the raised profile of QSARs in these frameworks, a gap exists in the evaluation of QSAR use and QSAR documentation under authorization. An assessment of the different uses (e.g., WoE and ITS) in which QSAR predictions play a role in evidence gathering and organizing remains unaddressed for AoA. This study approached the disparity in information for QSAR predictions by conducting a substantive review of 24 AoA through May 2017, which contained higher-tier endpoints under REACH. Understanding the manner in which applicants manage QSAR prediction information in AoA and assessing their potential within ITS will be valuable in promoting regulatory use of QSARs and building out future platforms in the face of rapidly evolving technology while advancing information transparency.

## 1. Introduction

Under REACH, quantitative structure–activity relationship (QSAR) models, which predict toxicological endpoints from a set of experimental values [1], play an increasingly larger role in providing vital physicochemical property and environmental and health hazard data to meet information requirements. Under registration, registrants may use QSARs as part of their hazard assessments, and they should be considered before performing vertebrate animal testing [2]. For REACH authorization, QSAR data have been especially useful in closing data gaps [1,3].

Evaluation of Registration, Evaluation, Authorization, and Restriction of Chemicals (REACH) QSAR use, however, has been solely directed at registration dossiers. Under registration, QSAR use contributes to a larger trend of non-testing methods. Significant “quality deficiencies,” however, have been noted in registrants’ use of alternative methods [4].

Despite an absence in regulatory reporting, QSAR use continues to trend under authorization. Under authorization, companies seeking continued use of their Annex XIV substance must examine suitable replacements or alternatives for problematic Annex XIV substances [5]. Due to limited experimental data on these alternatives, QSAR predictions may be the only information provided in the hazard assessment component of the application or analysis of alternatives (AoA) [3]. Furthermore, applicants use or may potentially use QSARs in ways that are unique to their applications for authorization. For instance, applicants may embed valuable information regarding their QSAR predictions within their AoA despite missing (Q)SAR Prediction Reporting Format (QPRF) documentation. Although previous work has analyzed fundamental QSAR uses in AoA [6], no assessments have been made which assess the different uses by which QSAR predictions factor into more complex contexts.

In response to the data gap, this study explores three novel uses of QSAR information in REACH authorization: (1) QSAR data, which falls under QPRF, (2) QSAR usage in weight of evidence (WoE), and (3) QSAR battery test integrated systems (ITS). This study begins with an assessment of select QPRF template criteria set out under the European Chemicals Agency (ECHA) guidance [1]. Using select QPRF criteria from ECHA’s QPRF guidance, this study conducts a careful examination of AoA with missing QPRF documentation to identify QPRF criteria within alternative chemical profiles.

As WoE remains unexplored in AoA, this study makes a comparative analysis on WoE completeness when using QSARs. To compare WoE, a comprehensive WoE framework was established, which drew from multiple regulatory WoE approaches to set out procedural criteria for WoE “completeness”. QSAR use within WoE in the AoA sample was then measured against this new framework.

Finally, even though ECHA encourages advanced testing strategies such as ITS [4], at the time of this study no evaluations have been performed on REACH AoA that explore the potential benefits of battery QSAR tests, especially for higher-tier endpoints. As this study expressly sets out to explore ITS in QSAR use on higher-tier endpoints, the Danish EPA QSAR battery test data were used as the basis of comparison. While the Danish EPA performed battery tests on a variety of endpoints, this study only referred to higher-tier endpoint data.

This paper presents a rigorous assessment of use of QSAR information in REACH AoA. An overview of the public and regulators’ need for sweeping QSAR information in REACH AoA is first presented, highlighting the need for fundamental changes in the approach to understanding QSAR use in REACH AoA. A detailed description of the methodology then follows. In all cases, QSAR use in AoA was analyzed for functionally equivalent information to a QPRF document as well as WoE regulatory standards and best practices in using QSARs in AoA. Since human and environmental endpoints hold the most relevance in regulatory discussions, this study focused on QSAR predictions for hazard endpoints.

### The Need for Heightened Transparency in REACH AoA QSAR Data

Detailed and open QSAR information is important for regulatory decision making, especially within the context of authorization, where alternatives may lack experimental data and applicants may need to rely on predictions for assessing toxicity. QSARs differ from other non-testing methods by quantitively estimating the toxicity of a target chemical [7,8]. In certain cases, for some in silico models, including QSARs, prediction accuracy exceeds animal tests [9]. As QSAR models extrapolate from test sets, which also carry inherent uncertainties [10], predictions are built on a set of assumptions. To carry out their hazard assessments, applicants are required to address these uncertainties in their AoA (Q)SAR documentation [1].

However, limited information exists that examines and reports the roles of QSAR use in AoA, leaving open the possibility of an applicant’s missteps going unrecognized and unreported to EU stakeholders. To our knowledge, no REACH reports exist that assess QSAR use in AoA. Furthermore, no reports have been issued that analyze advanced or alternative optimization of QSAR information in AoA. Examples of similar registration reporting breakdowns have already been noted in a variety of high profile evaluations for registration dossiers. A 2017 report by ECHA found “poor documentation,” and a “lack of qualitative and quantitative data to support predictions based on toxicokinetics” [4]. More generally, in reports by the German Federal Institute for Risk Assessment (BfR), REACH, and NGOs, the level of reporting and quality of information in registration dossiers was found to be “insufficient” [11]. Periodic evaluation of applicant QSAR use for oversight and quality control is therefore an equally important task for ensuring rigorous regulatory decision making under authorization. For stakeholders involved in an application’s public consultation, the apparent lack of regulatory reporting on QSARs in AoA poses a significant barrier to AoA transparency and quality assurance.

Examining QSAR predictions in AoA to meet information requirements is especially important when considering additional non-testing information requirements. For cases where QSARs are used as part of weight of evidence (WoE), or the combination of “multiple different experiments that purport to model a given endpoint … to give a stronger classification” [12], applicants need to provide well-documented QSAR predictions as well as complete WoE in order to fulfill information requirements. However, registration WoE guidance [13] has been noted as “short-spoken,” and lacking a systematic approach [14], which could open up too broad an interpretation of WoE and increase inconsistency in combining evidence to assess an endpoint or property. This is relevant for authorization, for which this guidance was not written. Moreover, critics note a lack of detailed WoE sub-criteria in regulatory guidance as well as an undue amount of importance placed on expert judgment [14]. Without proper monitoring of both of these efforts, a regulator may be unable to get a detailed enough explanation of how an applicant selected their information or what sub-criteria they used to make their decisions on the main criteria.

WoE is limited though and may not always give the best outcome on its own. In some cases, even if an applicant’s WoE presents an articulate, well-documented argument with a non-integrated battery of QSAR predictions, an applicant may still select the wrong QSAR model or approach. An integrated screening approach, on the other hand, combines tests based on battery testing or tiered testing and decision analysis, and is known to produce more powerful results [15]. According to Worth [16], an ITS approach is compatible with the use of QSAR predictions because QSAR models share many of the ITS principles. Combining predictions in an ITS approach, or what Hartung et al. [17] refers to as “Integrated Non-Testing Strategies” (INTS), has also been shown to increase prediction reliability and consistency [18] and decrease false positives [19] and “noise” [20]. Despite recent assessments stressing caution in the manner in which test results get integrated [15,21], particularly for ITS methods focusing on high sensitivity which may “overpredict toxic potential” [22] and lead to an “accumulation of false-positives leads” [17,23], the potential benefits of using QSARs in ITS methods, especially for priority higher-tier chemicals in AoA, remain unknown. WoE and ITS strategies are especially important for substances deficient in experimental data.

Harmonized classifications for carcinogenic, mutagenic, or reproductive toxicity (CMR) substances and persistent, bioaccumulative, and toxic or very persistent and very bioaccumulative (PBT/vPvB) substances, which are also identified as substances of very high concern (SVHCs), may, in fact, lead to SVHCs becoming a candidate for the Annex XIV list under authorization [24]. REACH has issued guidance on how to use ITS to meet registration information requirements and directs registrants to consider non-testing results, including QSAR predictions, when deciding if further animal testing is needed [25,26,27]. However, any recommendations or explanation of the comparative benefits of QSAR battery tests are not mentioned in their 2017 documents on endpoint-specific guidance nor has any guidance been issued for applicants under authorization. Nevertheless, reports on the regulatory use of QSAR battery tests have been circulating in the European Union (EU) for almost two decades. Since 2001, the Danish EPA has published their advisory hazard classifications list, which includes possible CMR endpoints, based on integrated QSAR results to supplement the vast number of unclassified substances in the EU market under which substances must meet strict standards to achieve advisory classifications (Supplementary S1). Without assessing the critical role of QSAR battery tests in information gathering and testing assessments for CMR and PBT/vPvB substances in AoA, less robust, individual model results may factor into authorization decisions despite the existence of potentially more reliable ITS predictions. This risk in regulatory decision making ultimately highlights the need for more attention to be placed on QSAR use in authorization.

## 2. Materials and Methods

### 2.1. Assessing Different Use of QSAR Data under Authorization for Increased Openness and Accuracy

#### QPRF Criteria

To investigate whether applicants provided information required under a QPRF, this study developed QPRF criteria using ECHA’s QPRF template. The ECHA QPRF template, made up of substance, general information, prediction, and adequacy main criteria, served as the baseline for standard QPRF information [1]. For substance information, chemical identifiers were surveyed using AoA data from a previous study of REACH AoA [6], coding for Chemical Abstracts Service (CAS) number, European Community (EC) number, chemical name, structural formula, and structure codes or text representations of a chemical’s structure. For prediction information, model identifiers such as the model’s hazard endpoint and dependent variable [1], model name and version, predicted value, input for the model used to generate the prediction such as the specific structure codes, and descriptor values which code molecular value into numerical value were reviewed and coded [28] (Table 1). Since this study incorporated previous research, which indicated that all QMRFs were missing, no QSAR Model Reporting Formats (QMRF) references in the QPRF nor any information relevant to the QMRF were coded.

As foundational information for a QPRF prediction plays a pivotal role in helping ECHA make an informed decision on the toxicity and safety of alternatives relative to the Annex XIV substance, QPRF “priority” criteria were highlighted for more in-depth analyses and in alignment with ECHA QPRF criteria [5] and Organization for Economic Cooperation and Development (OECD) Principles [29]. For this study, QPRF “priority” criteria are defined as foundational criteria, which provide fundamental information a regulator needs to draw a general conclusion on a QSAR prediction. The six, essential QPRF “priority” criteria include: (a) predicted value (ECHA QPRF criteria 3.2 and OECD Principle 2); (b) model endpoint (ECHA QPRF criteria 3.1 and OECD P Principle 1); (c and d) applicability domain and structural analogues (ECHA QPRF criteria 3.3 and OECD Principle 3); (e) uncertainty (ECHA QPRF criteria 3.4 and OECD Principle 4); and (f) chemical and biological mechanisms to conduct a more in-depth analysis (ECHA QPRF criteria 3.5 and OECD Principle 5) (see Table 1).

**Table 1:** 17 QPRF criteria ^†^, definitions and coding.

Category	QPRF Criteria	Definition	Description of Codes
*Substance information*	CAS number	American Chemical Society Chemical Abstracts Service Registry Number ^®^ (CAS RN^®^) identifier of up to 10 digits for each unique substance ^1^	0 = no CAS number reported
			1 = yes CAS number reported
			2 = non-applicable (N/A) i.e., there is no QSAR used for the alternative under consideration for a hazard endpoint
	EC number	European Union European Community (EC) seven-digit number used for the EC EINECS (European INventory of Existing Commercial chemical Substances), ELINCS (European LIst of Notified Chemical Substances), and NLP (No-Longer Polymers) inventory ^2^	0 = no EC number reported
			1 = yes EC number reported
			2 = N/A i.e., there is no QSAR used for the alternative under consideration for a hazard endpoint
	Chemical name: IUPAC and CAS names	International Union of Pure and Applied Chemistry and Chemical Abstracts Service chemical nomeclature ^3^	0 = no chemical name (IUPAC or CAS name) reported
			1 = yes chemical name (IUPAC or CAS name) reported
			2 = N/A i.e., there is no QSAR used for the alternative under consideration for a hazard endpoint
	Structural formula	Molecular formula showing chemical bonds and atom placement ^4^	0 = no structural formula reported
			1 = yes structural formula reported
			2 = N/A i.e., there is no QSAR used for the alternative under consideration for a hazard endpoint
	Structure codes (codes recognized by modeling software)	Simplified molecular-input line-entry system (SMILES) and IUPAC International Chemical Identifier (InChITM) identifiers using string representation of chemicals for computerized uptake by various electronic software programs ^5^	0 = no structural information for the substance, including identifiers, reported
			1 = yes SMILES identifier reported
			2 = yes InChI identifier reported
			3 = yes other identifier reported
			4 = yes substance is a stereo-isomer
			5 = N/A i.e., there is no QSAR used for the alternative under consideration for a hazard endpoint
*Prediction information*	Endpoint *	Different types of health impacts or different effects to the environment (ECHA 2011) ^6^	0 = no endpoint defined for which the model provides predictions
			1 = yes endpoint definedfor which the model provides predictions
			2 = N/A i.e., there is no QSAR used for the alternative under consideration for a hazard endpoint or the QSAR prediction was for physico-chemical endpoint
	Dependent variable	Biological activity predicted by QSAR model ^7^	0 = no dependent variable reported
			1 = yes dependent variable reported
			2 = N/A i.e., there is no QSAR used for the alternative under consideration for a hazard endpoint
	Model or submodel name	Model or submodel used for prediction (ECHA 2008) ^8^	0 = no model and submodel name given
			1 = yes model and submodel name given
			2 = N/A i.e., there is no QSAR used for the alternative under consideration for a hazard endpoint
	Model version	Version number and/or date of the model and submodel (ECHA 2008) ^9^	0 = no version number and/or date of the model and submodel identified
			1 = yes version number and/or date of the model and submodel identified
			2 = N/A i.e., there is no QSAR used for the alternative under consideration for a hazard endpoint
	Predicted value *	Model result with units and alerts for expert systems (ECHA 2008) ^10^	0 = no version number and/or date of the model and submodel identified
			1 = yes version number and/or date of the model and submodel identified
			2 = N/A i.e., there is no QSAR used for the alternative under consideration for a hazard endpoint
	Predicted value	Prediction cut-off value(s) used for classification i.e., degree of response (ECHA 2008) ^11^	0 = no explanation for cut-off values given for classification if the result is qualitative (e.g., yes/no) or semi-quantitative (e.g., low/medium/high)
			1 = yes explanation for cut-off values given for classificationIf the result is qualitative (e.g., yes/no) or semi-quantitative (e.g., low/medium/high)
			2 = N/A i.e., there is no QSAR used for the alternative under consideration for a hazard endpoint
	Input for prediction	Type of input e.g., SMILES, mol file, graphical interface, used to generate prediction (ECHA 2008) ^12^	0 = no input specified for generation of prediction
			1 = yes input specified for generation of prediction
			2 = N/A i.e., there is no QSAR used for the alternative under consideration for a hazard endpoint
	Descriptor values	Calculated molecular properties,(e.g., molecular weight), for selection of training set data ^13^	0 = no values reported for numerical descriptors
			1 = yes values reported for numerical descriptors
			2 = N/A i.e., there is no QSAR used for the alternative under consideration for a hazard endpoint
*Applicability domain*	Domains *	Response and chemical structure space in which the model makes predictions with a given reliability (OECD 2007) ^14^	0 = no discussion on whether chemical falls in the applicability domain of the model
			1 = yes discussion on whether chemical falls in the applicability domain of the model
			2 = N/A i.e., there is no QSAR used for the alternative under consideration for a hazard endpoint
	Structural analogues *	Similar structures and/or structural fragments found in both chemical of interest and QSAR model training set ^15^	0 = no list of structural analogues presented
			1 = yes list of structural analogues presented
			2 = N/A i.e., there is no QSAR used for the alternative under consideration for a hazard endpoint
	Uncertainty of the prediction *	Assumptions, experimental data, model cross-validation(s), information on the AD, and/or statistics on prediction error ^16^	0 = no comment on the uncertainty of the prediction
			1 = yes comment on the uncertainty of the prediction
			2 = N/A i.e., there is no QSAR used for the alternative under consideration for a hazard endpoint or there is no information on the AD
	Chemical and biological mechanisms *	Numerical values representing the chemical properties (e.g., molecular weight, rotatable bonds) and molecular descriptors ^17^	0 = no discussion on the mechanistic interpretation of the model prediction
			1 = yes discussion on the mechanistic interpretation of the model prediction
			2 = N/A i.e., there is no QSAR used for the alternative under consideration for a hazard endpoint

^†^ Excludes the optional ECHA criteria to explain adequacy [1] * Priority criteria. ^1^ [30]; ^2^ [31,32]; ^3^ [33]; ^4^ [34]; ^5^ [35,36]; ^6^ [37]; ^7^ [38]; ^8^ [1]; ^9^ [1]; ^10^ [1]; ^11^ [1]; ^12^ [1]; ^13^ [39]; ^14^ [29]; ^15^ [8]; ^16^ [1,10]; ^17^ [39].

Under the first criteria, a QSAR model’s prediction and endpoint are needed to understand the measured impact of a substance on human health and the environment. QSARs, which are developed from a set of experimental values tested for a toxicological endpoint [1], differ from other non-testing methods by quantitively estimating the toxicity of a target chemical [7]. In regulation, a QSAR’s predicted value can estimate toxicity because it establishes the mathematical relationship between the target chemical’s structure and its activity endpoint or physicochemical property [8] while a reported endpoint demonstrates how a QSAR model is used. Both pieces of information fundamentally help a regulator understand the consequences of exposing the environment and human health to this substance.

While the prediction and endpoint describe a substance’s impact on health and the environment, a model’s applicability domain (AD), or the “response and chemical structure space in which the model makes predictions with a given reliability” [29], helps to determine a prediction’s reliability. An AD contains the numerical interpretation of chemical measurements such as Log K_ow_ and molecular representations [1], structural fragments, mechanistic domains, analogues, and other considerations [8]. To meet the threshold of what is considered within domain, a test compound must fall within this response space, which impacts a prediction’s reliability when a QSAR model gets updated [29,40]. As the AD creates a model’s boundaries for its test sets, the AD will inform what predictions can be considered as more reliable than others. Roy et al. [40] explains this pivotal role in developing a robust QSAR model:

“In the construction of a QSAR model, the AD of molecules plays a deciding role in estimating the uncertainty in the prediction of a particular compound based on how similar it is to the compounds used to build the model”.

Even though a model’s output is not necessarily reliable, (i.e., it depends on where the result falls within the AD), the AD theoretically addresses how similar the test compound is to the training set chemicals used to build the model, though this largely depends on the degree to which the developer takes this into consideration throughout the process of building the QSAR model [41]. Ultimately, a prediction’s reliability is a compromise between the degree of restrictions placed on an AD and the amount of chemicals for which a QSAR model can make predictions [1,29]. Despite the complexity in interpreting an AD, the AD is still central to understanding a prediction’s reliability.

Structural analogues can also help to establish a QSAR prediction’s reliability. Structural analogues are fragments or “functional groups” of a chemical of interest that show the structural relationship between the model’s experimental data and the substance of interest [9,12,39]. According to ECHA, identifying structural analogues as well as appropriate representation in a model’s training set increases prediction reliability [8]. As the reliability of an endpoint estimate may be impacted by the physicochemical properties of the structural analogue, understanding the physicochemical properties of a structural analogue becomes important in determining a prediction’s reliability [1]. Due to the importance placed on AD and structural analogues in determining prediction reliability, this study included both criteria.

In contrast to the AD and structural analogues, which address prediction reliability, an uncertainty analysis identifies the data inconsistencies, which introduce error in the prediction. According to QPRF requirement 3.4 and OECD Principle 4, an assessor must perform an uncertainty analysis to identify QSAR prediction uncertainties [1]. This analysis accounts for internal model validation to determine the training set response, and predictivity or external model validation while considering the AD. If pertinent, experimental data, model cross-validation(s), information on the AD, and/or statistics on prediction error should also be provided [10]. Furthermore, uncertainty analyses should follow the “principle of precaution,” which, within the context of QSAR modeling, measures error between QSAR information input and output as well as any assumptions [1]. QSAR predictions, in particular, are characterized by “extrapolation uncertainty, which according to Sahlin’s [10] quote of ECHA is the “uncertainty involved in specification of numerical values” where model validation and in domain predictions need to be identified and considered. Identifying QSAR prediction uncertainties is especially important because predictions are based on a set of statistical assumptions. Thus, identifying any factors that contribute to the overall uncertainty of a prediction helps to establish “confidence” in non-experimental data for regulatory decision making [10] that impacts human health and wildlife [18,40,41].

Providing information on a model’s mechanistic processes, on the other hand, helps to define the appropriateness of the model to make predictions [1]. Unlike the mode of action (MOA), which identifies the key events leading to an effect [42], chemical and biological mechanisms make up the molecular processes underlying the key events [43]. Under QPRF criteria 3.5 and OECD Principal 5, these molecular processes can be extrapolated from the numerical values representing the chemical properties (e.g., molecular weight and rotatable bonds) and molecular descriptors or “predictors” to select the training or experimental data sets used to build QSAR models [1,39]. Ultimately, the appropriateness of the model for a specific chemical should be determined by comparing model and prediction output to existing experimental observations [OECD 29] though relevant models can be used for “hypothesis testing” to identify possible causal relationships [ECHA 1]. For more novel endpoints that involve complex mechanisms, however, interpretations of QSAR mechanisms may be unable to draw on existing scientific literature [7].

### 2.2. WoE Completeness Review

This analysis began with a “completeness review” in response to ECHA’s WoE guidelines. ECHA defines completeness as “whether the information is sufficient to make the regulatory decision” [1]. According to this guidance, a complete assessment of information supporting (Q)SAR results for regulatory decision making must be provided in addition to information on the relevance and reliability of the model and prediction. In this evaluation, “completeness review” is defined as a critical evaluation on the process and steps applicants took to formulate their WoE analysis for CMR and PBT/vPvB higher-tier endpoints when including QSAR predictions. The WoE completeness review began with an evaluation of REACH’s approach to WoE criteria set out in the 2016 *Practical guide: How to use alternatives to animal testing to fulfil your information requirements for REACH registration*. Criteria included assembling information that factored in relevance; reliability, adequacy, and quantity; discrepancies in studies; proper documentation; expert judgement; and “robust” summaries [13]. However, in applying this approach to this study, we identified inconsistencies and gaps in ECHA’s guidance similar to the gaps identified by Ågerstrand and Beronius for REACH WoE guidance [14].

As ECHA does not define “pooling” in their recommendation to “pool” information, nor identify steps on how to weigh the evidence or specify what to consider during data integration, this study drew from the 2018 National Resource Council’s (NRC) evaluation on the U.S. EPA IRIS system, and best practices from Rhomberg et al.’s 2013 review *A survey of frameworks for best practices in weight-of-evidence analyses*, Martin et al.’s 2018 review *Weight of Evidence for Hazard Identification: A Critical Review of the Literature*, and Suter et al.’s 2017 *A Weight of Evidence Framework for Environmental Assessments: Inferring Qualities*. These materials address three main deficiencies in ECHA’s WoE guidance: (a) creating broader WoE guidance to apply to other parts of REACH, specifically under authorization, (b) providing accepted metrics for “weighing” evidence; and (c) setting out specific steps for data integration. These frameworks and best practices form a comprehensive set of criteria for a transparent and structured application of WoE approaches (Supplementary Table S2) [44,45,46,47].

#### WoE Criteria Checklist

**Figure 1:**
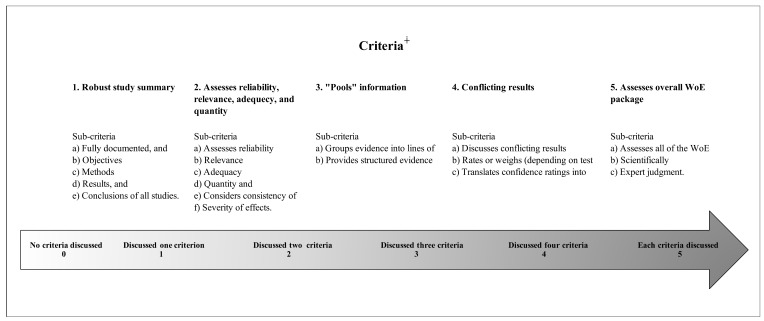
Checklist with quality scale for WoE completeness review. ^∔^ Criteria based on multiple frameworks and best practices [1,2,3,4].

Based on these comprehensive criteria, this study developed a checklist to conduct a “completeness review” for how well AoA articulated their WoE analysis when applied to higher-tier hazard endpoints (Figure 1). More specifically, this study focused on measuring the completeness across WoE using QSARs to gauge the degree to which applicants’ efforts met the WoE criteria. Completeness was rated on an increasing scale ranging from 0 = applicants did not discuss criteria to 5 = applicants discussed all criteria. When QSAR endpoint data were not applicable to the WoE completeness review, for example, when AoA had missing QSAR values, these data points were coded as “6” for non-applicable. Due to the scope of this study, the completeness review was limited to a procedural analysis and did not address substantive questions related to the quality of higher-tier WoE using QSARs.

Robust Study Summaries. For this checklist, the “objectives, methods, and conclusions” from REACH registration guaidance on the online Robust Study Summaries (RSS) acted as a checkpoint for each supporting material provided in an AoA WoE analysis. According to ECHA, an RSS [48] is a “detailed summary of the objectives, methods, results and conclusions of a full study report providing sufficient information to make an independent assessment of the study minimizing the need to consult the full study report” (Article 3 (28) of REACH).

As part of the WoE, REACH registrants provide an RSS in the technical dossier for each key study used [13]. Furthermore, to meet ECHA’s WoE criteria, a registrant must provide sufficient evidence; thus, more than one ESR can be included as well as supplemental proper documentation [13]. This study similarly examined whether applicants addressed objectives, methods, and conclusions and provided full documentation for test study results [13].

Reliability, Relevance, Adequacy, Quantity. The checklist’s second criterion explores whether a WoE analysis establishes the reliability (quality), relevance (appropriateness), and adequacy (usefulness) of existing studies that fall under WoE evidence selection. In the 2016 practical guide for how to use alternatives for testing, ECHA references Klimisch et al.’s [49] criteria and scoring methods. For each study, registrants give a Klimisch score ranging from 1 = reliable without restriction to 4 = not applicable to determine study reliability [13]. Due to the importance the different advisory and regulatory agencies [45,50] placed on these WoE principles, this study adopted Klimisch et al.’s criteria for reliability for this checklist. However, the Klimisch scoring method, which has been criticized for lacking detailed “criteria” and “guidance” as well as being biased towards standard practices, was excluded [51]. ECHA also requires companies to gather all available information on the chemical [13]. This checklist therefore added “quantity” to the checklist as a sub-criterion to add rigor and transparency. In addition, because this completeness review covers higher-tier endpoints, such as CMRs, which have chronic and acute dose effects on human health and the environment, this study included consistency of results as well as severity and type of effects towards this checkpoint.

“Pools information”/Lines of evidence. In the absence of an organizational framework for assembling this information, ECHA’s WoE guidance, Rhomberg et al. [47], and Martin et al.’s [44] reviews on the WoE framework and the National Resource Council’s best practices [45] served as the basis for the next criterion, “pools information”/lines of evidence”. According to Martin et al. [44], lines of evidence (LOE) are a useful grouping tool for similar information when assessing a substance’s hazard. Structured tables can also help to present evidence. Likewise, the National Resource Council (NRC) advises using structured tables so that different types of information can be organized into “individual data streams” that connect to the areas of studies [45]. Since WoE draws on multiple LOEs for integration, both “LOE” and “structured tables” were added to the third criteria of the checklist. This evaluation similarly adopts the NRC’s conclusions that few chemicals have accompanying mechanisms of action (MOA). Therefore, MOAs were excluded from this step of data integration [45].

Conflicting results. Lines of evidence, however, can contain conflicting results; therefore, differing lines of information need to be considered in a weighted manner [47]. In this checklist, “conflicting results” considers the question of relative weight or strength of evidence. As ECHA did not provide further steps for rating and weighting these results aside from stating that high quality in vivo and in vitro results and studies should receive greater weight than QSAR results [13], this study drew from multiple assessments. Suter et al. [46] recommend the use of scoring tables to build a more comprehensive WoE framework. In this assessment, a scoring table, which is based on general criteria such as “reliability” and “strength” of information, applies weighting using symbols such as “+, −, 0” to test the hypothesis on the chemical [46]. For this checklist, a more flexible version of Suter et al.’s scoring table was adopted; any table that indicated and/or compared hazardous endpoints was accepted. Furthermore, results were accepted if an applicant explained any ambiguities and discrepancies [46]. As a regulator must be able to effectively infer the alternative chemical’s impact, the inference of any health effects from weighting was added to the checklist [44]. Differences and inconsistencies in information as well as risk factors, such as “uncertainty” and “bias,” and “rigor” and “cohesion” in data integration across studies [47], additionally impact the integration of results. This study thus factors in “decision logic” challenges.

Final assessment. All evidence, which has been carefully classified and weighed, needs to be integrated into a final assessment based on expert judgment. ECHA notes that a WoE expert must have knowledge in the “relevant endpoints” and “study methods,” and must be able to make scientific judgments [13]. Rhomberg et al. [47] describe a WoE expert as someone who is specialized in toxicology, epidemiology, or methodology. Yet, ECHA does not lay out any expectations as to how this expert should be identified in a REACH AoA. While “prescriptive reporting templates” have been discussed as a way to systemize collective expert judgement, this type of large-scale regulatory change is beyond the scope of this paper [44]. For this study, the “Conclusions” and “Reduction of Overall Risks” sections represent this final assessment, which were coded as “Assess overall WoE package”. To determine whether expert judgment was used, this study examined any detailed discussion in the form of a conclusion that considered the reliability, relevance, and adequacy of WoE information, which has been integrated and compared, and assigned a weight to each piece of data [13]. For this criterion, the extent to which applicants drew conclusions on the safety of the alternative relative to the Annex XIV chemical was examined.

For this study, any WoE observations were recorded for each of the five main criteria (Figure 1) in Libre Office Version: 6.2.4.2. Based on the descriptive statistics, assessments looked at the degree to which applicants met the criteria for a rigorous WoE analysis. Sub-criteria for each of the five main criteria were carefully tracked and recorded. To maximize the small sample size and limit random error, a baseline of >60% fulfilled sub-criteria was used to satisfy each main criterion. For all descriptive statistics, “alternative per AoA consultation number” served as the unit of analysis. Taken together, there were a total number of 54 opportunities per alternative per consultation number to provide information for criteria, which reflected the number of times an AoA applicant could have submitted a complete WoE that included QSAR predictions.

### 2.3. ITS QSAR Comparative Analysis

Using the Danish EPA advisory list, this study compared individual QSAR model predictions from 24 AoA against Danish EPA battery test predictions for CMR substances. The intention of the approach is to assess the similarity of results and examine the potential benefits of integrated QSAR results.

#### Danish EPA Advisory List

Despite the potential advantages associated with using battery QSAR test results, no other research has investigated the value of ITS in REACH AoA that cite QSAR predictions. This study approached the most recent 2018 Danish EPA advisory classification results with a comparison of all assessments of QSAR predictions from our original sample AoA (*n* = 24). To confirm the accuracy of applicant information, this assessment began with a verification of Chemical Abstracts Service (CAS) numbers, which are unique numbers assigned to chemicals used in the science field [30] (Table 2). (For more information on CAS number verification, see Supplementary S1).

Once the identity of each alternative was confirmed, AoA CAS numbers were compared with CAS numbers in the Danish EPA advisory list using Excel (Version 16.28) [52]. For any matches, information for the ITS battery QSAR prediction advisory classification were recorded in an Excel spreadsheet: Muta. 2 (Suspected of causing genetic defects); Carc. 2 (Suspected of causing cancer); or Repro. 2 (Suspected of damaging fertility or the unborn child). CMR endpoints are defined under the Danish EPA’s battery of model endpoints (Table 3). AoA with matching CAS numbers were visually inspected for any CMR identifiers for the alternative. The following sections received the primary focus: Mammalian hazard profile, Reduction of Overall Risk, Conclusion on suitability and availability, Comparison of hazards, and the Annex. Any supporting, conflicting, or missing information was reported separately. A discussion on the likely impact of a consolidated ITS framework on alternative hazard assessments concludes this assessment. In this paper, an integrated approach to QSAR use was considered, when appropriate, to be forward looking.

## 3. Results

### 3.1. QPRF Criteria

**Figure 2:**
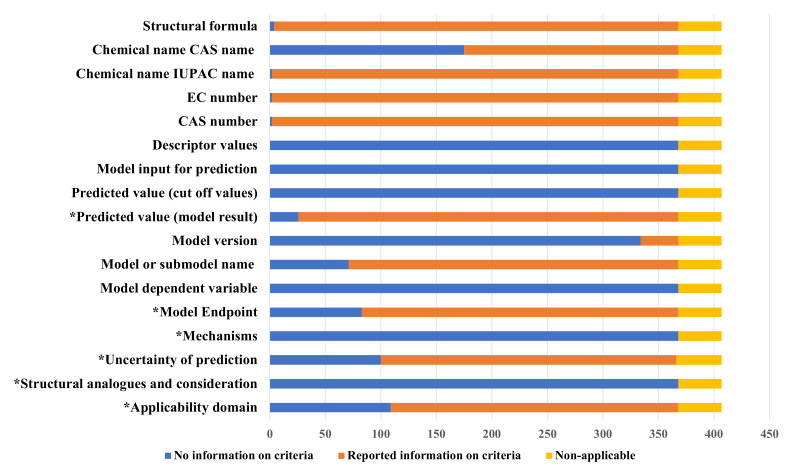
Collected descriptive statistics on 16 QPRF criteria. * QPRF “priority” criteria.

Overall, criteria were unevenly distributed (Figure 2). No information was identified for the model’s dependent variable, cut-off values for the prediction, model input for the prediction, structural analogues, and biological mechanisms. However, all QSAR predictions provided information on the structural formula, International Union of Pure and Applied Chemistry (IUPAC) name, and EC and CAS number criteria of their alternative. For chemical structure codes, (i.e., the 17th criterion), only Simplified Molecular Input Line Entry System (SMILES) codes were provided.

**Figure 3:**
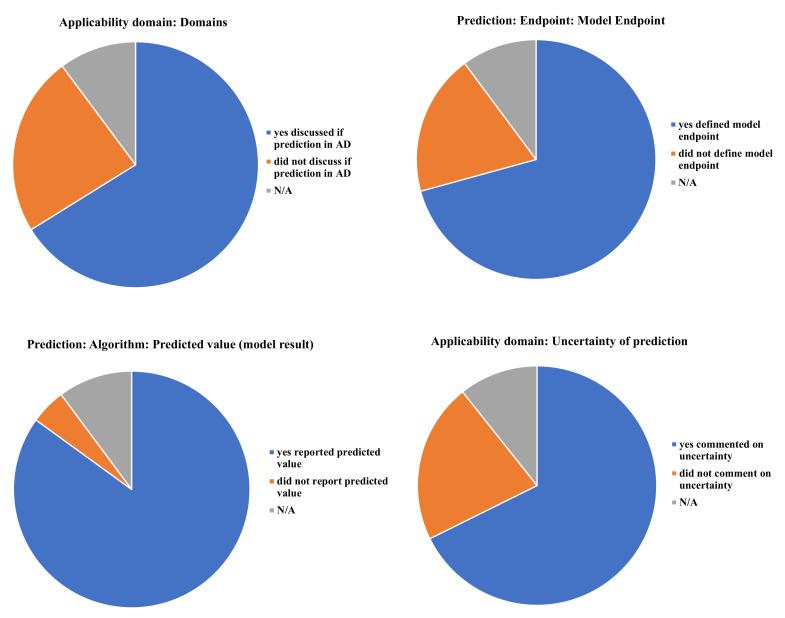
Four priority criteria for analysis.

Results for the four priority QPRF criteria were more evenly distributed (Figure 3). For the applicability domain: predictions were discussed in reference to their applicability domain 260 times while predictions were not discussed in the context of their applicability domains 93 times. While applicants commented on the uncertainty of predictions 266 times, they did not comment on a prediction’s uncertainty 85 times. Under “uncertainty”, no information was given on the AD two times. Though predicted values were cited the majority of the time (*n* = 334), qualitative or quantitative predicted values were identified as going unreported 19 times. The QSAR model endpoint was defined, at least partially, 278 times, though applicants did not report the model endpoint for 75 predictions. However, no information was given for either structural analogues or chemical and biological mechanisms.

The model endpoint description priority criterion had the least detailed information. On the one hand, some model endpoint descriptions had better details than others. For example, consultation number 0005-02 for methyl centralite for genetic toxicity reported: In vivo–Mutagenicity, QSAR prediction for Rodent dominant lethal assay from the Danish (Q)SAR Database (DQD). For this AoA, the applicant, DEZA a.s., indicated both the specific assay and animal testing in addition to the hazard endpoint. However, most endpoint descriptions did not contain the exact model endpoint nor the experimental test. For the alternative ethyl centralite, also from consultation number 0005-02, DEZA a.s. simply noted EPI Suite (BCFWIN) bioaccumulation potential: Log BCF (predicted by BCFWIN) [53] ethyl centralite, without indicating if the result was aerobic or anaerobic biodegradation [54].

### 3.2. AoA That Used QSARs in WoE for Higher-Tier Endpoints and Completeness Review

Of the 24 AoA, only three used QSARs for at least one higher-tier endpoint. These AoA performed WoE for higher-tier endpoints on 11 unique alternatives (Table 4). As data were analyzed by the unit of alternative by consultation number, the same alternative may have been assessed multiple times by different applicants. For example, consultation numbers 0005-01 and 0006-1 both assessed the alternative diisobutyl hexahydrophthalate (DIBE).

**Figure 4:**
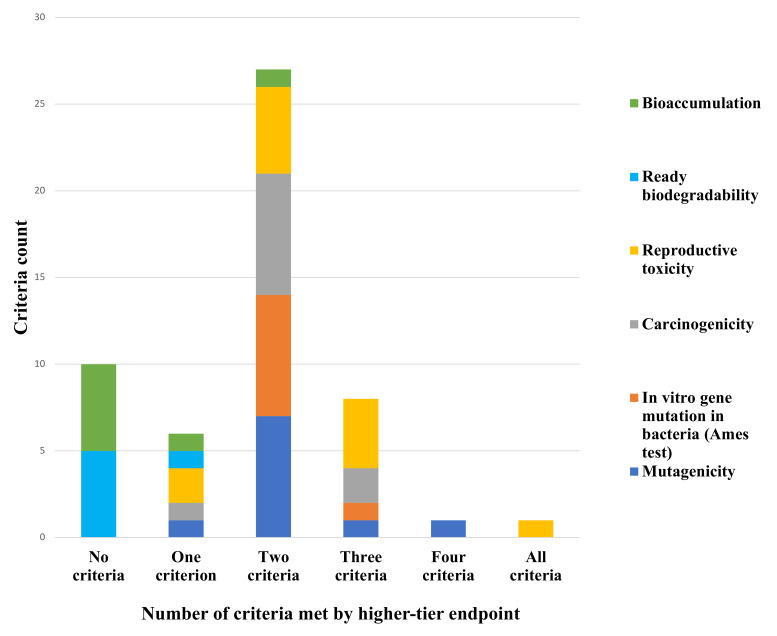
Number of criteria met in criteria checklist by QSARs used in WoE sub-divided by higher-tier endpoints. Notes: (a) Robust study summary: fully documented. Includes objectives, methods, results, and conclusions of all studies. (b) Assesses reliability, relevance, adequacy, and quantity. Considers consistency of results and severity of effects. (c) “Pools” information by grouping evidence into lines of evidence and providing structured evidence tables. (d) Conflicting results: Rates or weighs (depending on test method, data quality, and endpoint) using scoring table, and translates confidence ratings into level of evidence for health effect. (e) Assesses overall package. Scientifically justified/argued using expert judgment.

QSARs used in WoE to assess CMR endpoints (*n* = 40) varied in quality of completeness for the five main criteria. In this analysis, quality of completeness was assessed across three consultation numbers and 12 different alternatives. However, only one WoE analysis (assessment of reproductive toxicity of the alternative bis(2-ethylhexyl) adipate (DEHA) in consultation number 0005-02) met all five WoE criteria (Table 5). In the same AoA, the WoE analysis for mutagenicity for the alternative Akardite II met four of the criteria: (1) “Pools” information; (2) Conflicting results; (3) Assesses reliability, relevance, adequacy, and quantity; and (4) Assesses the overall WoE package. The WoE analyses for the alternatives Akardite I, ethyl centralite, and methyl centralite met the least amount of criteria for ready biodegradability, which indicates rapid breakdown in most environments [55], and bioaccumulation. The WoE analysis for dioctyl azelate (DOZ), on the other hand, met two criteria for bioaccumulation: (1) Assesses reliability, relevance, adequacy, and quantity; and (2) Assesses overall WoE package. Figure 4 illustrates a high-level view of these trends. In this figure CMR/PBT vPvB endpoint data are consolidated and organized by the number of criteria met. 

### 3.3. ITS Comparative Analysis

Most AoA CAS numbers did not have a matching CAS number in the Danish EPA advisory list except for four CAS numbers: 103-23-1 (DEHA), 53306-54-0 (DPHP), 7790-7 (ATBC), and 77-94-1 (TBC). All matching Danish EPA advisory list CAS numbers had a Repr. 2 classification (Table 6). None of the matching alternatives were assigned Muta. 2 or Carc. 2 classifications.

AoA consultation numbers 0002-01, 0002-02, 0003-01, 0003-02, 0004-01, 0004-02, and 0005-02 identified reproductive toxicity for the alternative bis(2-ethylhexyl) adipate DEHA (Table 6). In each of the AoA Reduction of overall risk assessments, applicants reported CMR concerns for DEHA. Moreover, DEHA made the ECHA’s Community Rolling Action Plan (CoRAP) list [56] due to CMR concerns [53,57,58,59,60,61,62]. Teratogenicity was also cited in each of the AoA Annexes. In addition, applicants reported a Repro. 2 notified classification for DEHA in the “Notified classification and labelling of DEHA according to CLP criteria” tables [53,57,58,59,60,61,62]. Finally, consultation number 0005-02 cited uncertain reproductive toxicity in its AoA’s Comparison of Hazards Table 4.53 [53].

For consultation numbers 0002-01, 0002-02, 0003-01, 0003-02, 0004-01, and 0004-02 for the alternative bis(2-propylheptyl) phthalate (DPHP), there was no mention of reproductive toxicity in the Reduction of overall risk assessment (Table 7). Similarly, for the alternative acetyl tributyl citrate (ATBC), there was no mention of reproductive toxicity in the Reduction of overall risk assessment or the Comparison of Hazards tables for consultation numbers 0002-01, 0002-02, 0003-01, 0003-02, 0004-01, 0004-02, and 0005-02. However, for consultation number 0005-02, in the Mammalian hazard profile, the applicant did mention a reproductive toxicity effect dose above 300 mg/kg bw/d [53,63].

Finally, for the alternative tributyl citrate (TBC) in consultation number 0005-02, the applicant stated a lack of “documented data” on TBC’s reproductive toxic effects despite providing a negative QSAR prediction originating from the Teratogen Information System (TERIS) database in Table 4.68: Human health and environmental hazard profile for TBC (Table 7) [53].

## 4. Discussion

The evaluation of QSAR use in REACH AoA for default QPRF information, WoE completeness, and QSAR battery testing is necessary because QSAR predictions can impact an applicant’s conclusions in an AoA hazard assessment, and ultimately, authorization decisions. Authorization decisions are based on REACH AoA data; therefore, QSAR data used in AoA need to be transparent and rigorously monitored. However, at the time of this study, no regulatory system under REACH exists to monitor existing and innovative QSAR uses under REACH authorization. This study began by exploring transparency in REACH QSAR documentation and usage in AoA and three major points were identified: (a) QPRF data given in AoA especially for one priority criteria were insufficient and lacked in quality, (b) only a limited number of AoA used WoE with QSARs, and completeness varied depending on the main criteria and hazard endpoint, and (c) ITS QSAR battery models could provide significant benefit to the REACH AoA community.

### 4.1. QPRF Equivalency

Under REACH, applicants are required to submit QSAR data in a QPRF form. However, ECHA has yet to enforce QPRF documentation for REACH AoA despite the fact that QPRF information is vital to decoding the reasoning behind an applicant’s conclusion as to whether a prediction is acceptable under regulatory terms. As QPRFs were missing for all AoA in this study’s sample, data were collected by prediction/QSAR source/alternative for hazard endpoints and tabulated under 17 main QPRF criteria (Table 1). These criteria were considered fundamental for informing whether a QSAR’s prediction is reliable or not. From this review, several criteria were found not to have been met, including one of the priority criteria, structural analogues. The majority of applicants, however, provided prediction information for uncertainty, AD, predicted value, and model endpoint, though missing QSAR prediction information for the six criteria, (i.e., descriptor values, model input for prediction, prediction value, model dependent variable, mechanisms, and structural analogues) were identified in all 24 AoA.

Due to the large amount of missing QPRF information, questions still surround the degree to which a regulator would deem the existing information adequate if not fully sufficient. While an alternative stream of information was embedded in AoA, albeit informally and in incomplete form, including at least four of the priority criteria, a high degree of quality cannot also be assumed. For example, although qualitative QPRF predicted values such as positive/negative and semi-quantitative information, (e.g., less than 5 or between 5 and 10) were reported 192 and 30 times, respectively, they were given without their cut-off values for classification. Hence, a regulator might not be able to judge an alternative’s toxicity or safety without knowing the cut-off values offhand. In this study, missing cut-off values occurred for all qualitative and semi-quantitative QSAR predictions.

In addition, most model version information was missing. This became an issue when this study attempted to verify AD information. For the alternative methyl centralite in consultation number 0005-02 and the hazard endpoint “Genetic toxicity: In vivo—Chromosomal effect” for the mouse bone marrow sister chromosome exchange assay, the applicant used the DQD to report an “equivocal” result that was “within QSAR domain” [53]. Yet, when the same prediction was downloaded on 15 March 2019, an inconclusive result was generated from battery QSAR models, which indicated the prediction was out of domain [64]. Interestingly, the DQD reported a positive but out of domain result for each QSAR prediction, i.e., Leadscope, CASE Ultra, and SciQSAR. If both results could be verified as coming from the same model version, then the question of whether applicants cited QSAR results as in domain when they were, in fact, out of domain could be answered (Supplementary S1). On the other hand, it could simply be an isolated error. The DQD has been updated several times since the November 2015 publication [65]. Arguably, these errors may not make a difference in the outcome of the relevant AoA. However, it is an indication of possible deficient practices that could matter in later AoA.

The quality of reporting by applicants was also examined and found to be poor. For instance, applicants commented on a prediction’s uncertainty 266 times. However, these comments lacked depth and fell short of communicating the importance owed to a discussion on a prediction’s uncertainty. Specifically, many comments were single responses or short phrases such as “uncertain”, “acceptable,” or “doubtful reliability”. Poor reporting has been similarly noted in REACH registration dossiers [11].

At the same time, the QPRF template is in no way perfect. Suggested changes to the QPRF format during the 2nd European Union (EU) Technical Committee on New and Existing Chemical Substances (TCNES)/(Q)SAR Working Group meeting (January 2006) included creating more defined headings such as “other information regarding prediction reliability” to provide more useful information [66]. In addition, Walker et al. [67,68] recommended that confidence intervals accompany predictions, especially since descriptors are oftentimes generated by other QSARs, “thus increasing the potential for error propagation”. Regardless, the value of having a QPRF is evident, without which basic cases of ambiguity and equivocal language such as indicating four days for a chronic toxicity duration [69] or defining the model endpoint but also writing the prediction was for an undefined endpoint [53] cannot be resolved and could leave the regulator in a position of disregarding or misinterpreting the QSAR prediction altogether.

A final point of discussion is that without stricter regulatory oversight, major errors such as missing QSAR source/model names could go unchecked and perhaps lead to misinformed regulatory decision making on Annex XIV substances. In consultation number 0005-02, several PBT/vPvB QSAR predictions for the alternative tributyl citrate (TBC) had missing QSAR models/sources. When referencing information presented on the ECHA Dissemination Portal in the RSS for environmental fate and behavior and ecotoxicology, DEZA a.s. reported, “[I]t was found to have a calculated bioconcentration factor (BCF) of 94.7 L/kg wet-wt”. [53]. However, after querying the ECHA Dissemination Portal for (Q)SAR aquatic/sediment and terrestrial bioaccumulation factor calculations, TBC did not even show up as a result [70]. Likewise, in consultation number 0006-01, the applicant Sasol-Huntsman GmbH & Co. KG reported a series of OECD Toolbox predictions for bioaccumulation for the alternative DIBE [53]. However, the applicant did not identify the source of these predictions, nor could any QSAR predictions be found in consultation number 0006-01′s Table 4.2: Physicochemical properties of DIBE, or Table 4.5: Human health and environmental hazard profile for DIBE [53]. Notwithstanding, QSAR predictions of hydrophobic compounds present multiple challenges to generate accurate octanol–water partition coefficient (log P) predictions, which further underscores the need to know the QSAR model [71]. Thus, if ECHA wanted to confirm QSAR predictions or research any QSAR evidence based on the predictions, not only would they be unable to find this information in the AoA, other applicants who share information may replicate the same error, further lowering the quality of AoA. At the same time, approaches to addressing uncertainty need to be inclusive of forward-thinking regulatory use of QSARs not necessarily addressed in the OECD principles in addition to initiatives around state-of-the-art A.I.-based QSAR models [72,73].

### 4.2. AoA That Used QSARs in WoE for Higher-Tier Endpoints and WoE Completeness Review

Findings from the completeness review of 24 AoA, which assessed 54 non-unique alternatives, revealed that only a limited number of AoA used WoE with QSARs. In addition, WoE completeness varied depending on the main criteria and hazard endpoint. Additionally, applicants frequently failed to identify the sources of QSAR predictions in their WoE or did not address positive QSAR predictions in their WoE. For example, DEZA a.s. reported a positive biodegradation probability (i.e., Biodegradation = 0.0403) using an EPI Suite^TM^ BIOWIN MITI QSAR model prediction in consultation number 0005-02, for the alternative methyl centralite. This result indicates persistence [74]. However, this prediction was not included in the discussion on environmental fate and pathways toxicity [53].

Available information, based largely on the outputs of various QSAR models, does not raise concern for either the persistence or bioaccumulative potential of the substance in the environment.

Although there are other regulatory and best practice cut-off points for a substance to be considered not-readily biodegradable, methyl centralite’s prediction of 0.0403, which is clearly in the range of not readily biodegradable, should have been addressed under the conflicting results WoE criteria. According to Posthumus et al. [75], a substance with a biodegradable score of 0.0403 is considered persistent. In addition, Posthumus et al. reported that for the EPI Suite^TM^ BIOWIN MITI models, for a substance to be considered persistent, not only must the probability of a substance be <0.5, but the substance must also meet two other criteria:

- the probability of the non-linear rapid BIODEG model is <0.5, and
- the result of the ultimate survey model is <2.2.

As the applicant did not explain how they weighted biodegradation, this study could not conclude if DEZA a.s. factored this positive prediction for persistence into their conclusions. Ecological data supporting decisions of Environment Canada on methyl centralite (Table 4.8 in consultation number 0005-02) listed several predictions and experimental results for which any of the persistence and bioaccumulation data could have been part of the line of evidence for non-testing data before any weighting [53]. In the end, these results reveal the differences in WoE completeness among the applicable sample AoA with important implications for transparency in the use of QSARs in AoA. Moreover, these gaps in completeness provide insight into areas that need to be highlighted in future AoA WoE guidance that has implications for prioritization purposes even outside REACH [76].

**Figure 5:**
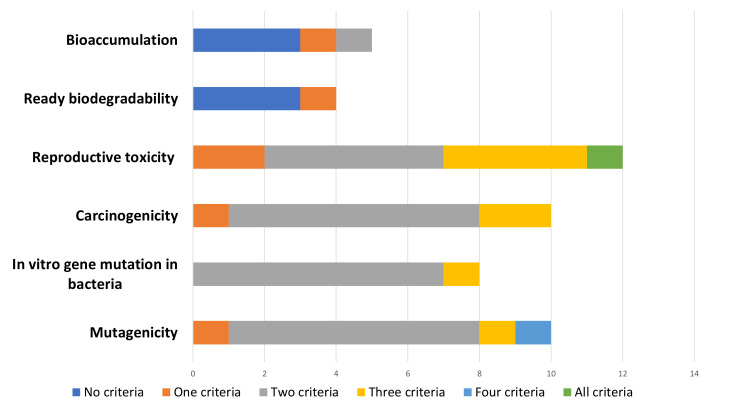
Differences in number of criteria met in criteria checklist by QSARs used in WoE by CMR/PBT vPvB endpoints.

Alternatively, the three AoA that did use WoE involving QSAR predictions for reproductive toxicity consistently met the majority of the five main criteria to a higher degree than the other CMR/PBT/vPvB endpoints (Figure 5). In fact, the findings on completeness for WoE using QSARs for reproductive toxicity demonstrate the progress made in bridging data gaps for this endpoint. In a 2011 article on reproductive and developmental toxicity in REACH dossiers, the author recommended more support for the development of non-testing methods for reproductive toxicity testing [77]. The article noted how data gaps for endocrine disruption also affected REACH information requirements. While REACH did not set out explicit guidance requesting information for this health endpoint, in the completeness review applicants provided the most information for WoE using QSARs under reproductive toxicity. One possible explanation for this occurrence could be the increased access to the freely available online QSAR models and QSAR predictions in the DQD. Alternatively, with REACH’s increased focus on higher-tier endpoints, AoA applicants may simply have been more diligent in providing this information.

One of the key takeaways of this analysis is that without greater monitoring of QSAR use in WoE for higher-tier endpoints in AoA, there could be significant implications for the final decision-making process. One such application that could help ECHA in monitoring WoE use in AoA and to make the process more standard and transparent could be the development of an online platform for WoE in authorization similar to the IUCLID registration software, which uniformly guides registrants through formatted prescribed fields [78]. Incorporating standardized online platforms such as the SciRAP tool could also help to define reliability for supporting material [51]. Grading of Recommendations, Assessment, Development, and Evaluation (GRADE) is another prescriptive approach, which uses a rating system to determine the “quality of evidence in systematic reviews and guidelines and grading strength of recommendations in guidelines” [79]. In fact, the NRC has already recommended this systematic approach to WoE for IRIS [80]. Finally, incorporating elements from a multi-criteria decision analysis-based (MCDA) approach may help standardize the assigned weighting of information beyond the Klimisch scores used for reliability. According to Linkov et al. [81], MCDA combines “value-based assessment” with expert decision making and scientific judgment by weighting the individual lines of evidence. Ultimately, these recommendations have the potential to combine all steps into one unified process, integrating “social, political, and economic considerations” into the WoE framework as a whole [81]. In the end, there would no longer be a need to separately analyze the technical and economic feasibility portions of the AoA. Moreover, building from an existing WoE platform for alternative testing will likely increase the amounts of properly completed WoE. However, ECHA must first provide the necessary guidance for authorization users in order for this to happen.

### 4.3. ITS Comparative Analysis

As ITS can be the precursor to evidence compiled in a WoE, this study also compared 24 AoA with the 2018 Danish EPA’s advisory list to screen for potential CMR substances that standalone QSAR may miss. This study revealed that ITS QSAR predictions can contribute to a broader understanding of a chemical in an alternative substance’s assessment. For instance, in AoA consultation number 0005-02, the QSAR predicted a negative result for potential teratogenicity for TBC [53]. However, the Danish EPA ITS battery QSAR model prediction for TBC predicted a positive response for reproductive toxicity [52]. Having a more powerful result from the Danish EPA could impact an applicant’s conclusion on the safety of TBC. Rather than drawing a conclusion of “no concern” for the hazard profile of an alternative based, in part, on a negative prediction for reproductive toxicity, an applicant, if informed by the Danish EPA ITS battery QSAR results, could conclude that there is some degree of concern for reproductive toxicity. For example, while the QSAR predictions in consultation numbers 0002-02, 0002-01, 0004-01, 0004-02, 0003-01, and 0003-02 for DEHA, DPHP, and ATBC indicated no reproductive toxicity, the Danish EPA assigned a Repr. 2 advisory classification to all three substances [57,58,59,60,61,62]. One possible explanation for this discrepancy could be that DPHP does not exhibit reproductive toxicity based on available studies of teratogenicity and reprotoxic effects at the highest doses. However, under Article 12(1) and Annex VI, companies are still required to report non-testing methods when appropriate [37].

Of course, merely recommending QSAR battery tests as individual or supporting evidence is insufficient to compel applicants to implement these types of tests into their hazard assessments. Strong regulatory assessment of AoA in the form of periodic reports is needed to actively track and enforce applicants to provide a detailed rationale for including or excluding data on alternatives that have hazardous implications. For example, even though the hazard class of Repr. 2 was shared among AoA for the alternative DEHA [57,58,59,60,61,62], in the end, the applicants did not factor reproductive toxicity into their conclusions. When noting the “slightly positive” response for the dominant lethal mouse assay, there is no mention of reproductive toxicity which, according to the Danish EPA, has the resulting effect of “early embryonic deaths” in the dominant lethal test in rodents [82].

Despite the potential advantage that QSAR battery tests have to offer, namely, strengthening the robustness of results, the comparison of alternatives with the 2019 Danish EPA advisory list illustrated the narrow regulatory use of either single or ITS QSAR models. Limited consideration of these QSAR models as appropriate tools is a repeating barrier to regulatory transparency. Despite the potential benefits of using QSAR battery tests, users should still remain critical of data inputs, training sets, and applicability domain thresholds, all of which can significantly affect model performance and prediction reliability [83]. In conclusion, ECHA should provide guidance on ITS QSAR models for authorization and other areas under REACH that frequently encounter data gaps. For example, to encourage regulatory acceptance, ECHA should draft guidance that includes ITS QSAR models such as the Danish EPA to meet information requirements under authorization. Similarly, the Danish EPA and ECHA could partner as change agents to develop an international ITS framework within the AoA community.

During this analysis, several assumptions were made to process the data in a uniform manner. First, multiple definitions of applicability domain (see Section 2 Methods: Data collection) were accepted. Regarding uncertainty of QSAR predictions, several versions were also accepted including “acceptable,” “limited similarity and no conclusion could be drawn,” “doubtful reliability,” “uncertain reliability,” “robustness of prediction,” “considered reliable,” and “no conclusion should be drawn”. Not accepted, however, were “no indication that model was operating outside of its operational limits,” as this interpretation did not answer the degree to which an applicant considered the prediction trustworthy. For QSAR source/model names, “OECD QSAR” was excluded as a source because this study’s approach could not confirm whether the applicant ran a QSAR model or if the QSAR prediction was cited from within the OECD (Q)SAR Toolbox, which would then mean it came from any number of QSAR models. Since OECD QSAR Toolbox houses a variety of sources and tools, which can generate different types of predictions, not all results identified as predictions could be categorized as QSAR predictions with any confidence [84]. Finally, partial endpoint definitions were accepted.

## 5. Conclusions

Identifying trends under REACH AoA in QPRF, WoE, and ITS frameworks, which draw on QSAR predictions, is the first step towards understanding the degree to which QSAR predictions fulfill regulatory expectations and play a role in driving these frameworks forward in 21st century toxicology. The results from examining AoA as an alternative avenue of QPRF information suggest that without the enforcement of QPRF documentation, regulators may be at a disadvantage when trying to access QSAR prediction information. AoA applicants in this study, on the other hand, prepared their AoA with limited formal guidance from ECHA on QSAR use. These AoA failed to provide any information at all for several criteria. In the end, results for meeting priority criteria showed that there is a baseline of information that one ought to know if submitting or assessing an AoA.

## Figures and Tables

**Table 2:** Alternatives from AoA sample (*n* = 24) for comparison with 2018 Danish EPA advisory list for self-classification of hazardous substances.

Alternative Substance	Consultation Number	CAS Index Name	Molecular Formula	CAS Number
(Listed in AoA)				
1,2,4-Trifluorobenzene	0078-01	Benzene, 1,2,4-trifluoro-	C_6_H_3_F_3_	367-23-7
Acetyl Tributyl Citrate (ATBC)	0002-01	1,2,3-Propanetricarboxylic acid, 2-(acetyloxy)-, 1,2,3-tributyl ester	C_20_H_34_O_8_	77-90-7
Acetyl Tributyl Citrate (ATBC)	0002-02	1,2,3-Propanetricarboxylic acid, 2-(acetyloxy)-, 1,2,3-tributyl ester	C_20_H_34_O_8_	77-90-7
Acetyl Tributyl Citrate (ATBC)	0003-01	1,2,3-Propanetricarboxylic acid, 2-(acetyloxy)-, 1,2,3-tributyl ester	C_20_H_34_O_8_	77-90-7
Acetyl Tributyl Citrate (ATBC)	0003-02	1,2,3-Propanetricarboxylic acid, 2-(acetyloxy)-, 1,2,3-tributyl ester	C_20_H_34_O_8_	77-90-7
Acetyl Tributyl Citrate (ATBC)	0004-01	1,2,3-Propanetricarboxylic acid, 2-(acetyloxy)-, 1,2,3-tributyl ester	C_20_H_34_O_8_	77-90-7
Acetyl Tributyl Citrate (ATBC)	0004-02	1,2,3-Propanetricarboxylic acid, 2-(acetyloxy)-, 1,2,3-tributyl ester	C_20_H_34_O_8_	77-90-7
Acetyl Tributyl Citrate (ATBC)	0005-02	1,2,3-Propanetricarboxylic acid, 2-(acetyloxy)-, 1,2,3-tributyl ester	C_20_H_34_O_8_	77-90-7
Akardite I (1,2-Diphenyl Urea)	0005-02	Urea, N,N’-diphenyl-	C_13_H_12_N_2_O	102-07-8
Akardite II (3-Methyl-1,1,-Diphenylurea)	0005-02	Urea, N’-methyl-N,N-diphenyl-	C_14_H_14_N_2_O	13114-72-2
Akardite III (3-Ethyl-1,1,-Diphenyl Urea)	0005-02	Urea, N’-ethyl-N,N-diphenyl-	C_15_H_16_N_2_O	18168-01-9
Benzene, Ethenyl-, Polymer With 1,3-butadiene, brominated (brominated co- polymer of styrene and butadiene) (Polymeric Flame Retardant (pFR)	0013-01	Benzene, ethenyl-, polymer with 1,3-butadiene, brominatedpolymer, manual registration, generic registration	N/A	1195978-93-8
Benzene, Ethenyl-, Polymer With 1,3-butadiene, brominated (brominated co- polymer of styrene and butadiene) (Polymeric Flame Retardant (pFR)	0013-02	Benzene, ethenyl-, polymer with 1,3-butadiene, brominatedpolymer, manual registration, generic registration	N/A	1195978-93-8
Chromium(III) chloride	0035-01	Chromium chloride	Cl_3_Cr	10025-73-7
Chromium(III) chloride	0036-01	Chromium chloride	Cl_3_Cr	10025-73-7
Chromium(III) chloride	0037-01	Chromium chloride	Cl_3_Cr	10025-73-7
Chromium(III) chloride	0038-01	Chromium chloride	Cl_3_Cr	10025-73-7
Chromium(III) chloride	0039-01	Chromium chloride	Cl_3_Cr	10025-73-7
Chromium(III) chloride	0040-01	Chromium chloride	Cl_3_Cr	10025-73-7
Chromium(III) chloride	0041-01	Chromium chloride	Cl_3_Cr	10025-73-7
Chromium(III) chloride	0041-02	Chromium chloride	Cl_3_Cr	10025-73-7
Di(2-ethylhexyl) adipate (DEHA)	0002-01	Hexanedioic acid, 1,6-bis(2-ethylhexyl) ester	C_22_H_42_O_4_	103-23-1
Di(2-ethylhexyl) adipate (DEHA)	0002-02	Hexanedioic acid, 1,6-bis(2-ethylhexyl) ester	C_22_H_42_O_4_	103-23-1
Di(2-ethylhexyl) adipate (DEHA)	0003-01	Hexanedioic acid, 1,6-bis(2-ethylhexyl) ester	C_22_H_42_O_4_	103-23-1
Di(2-ethylhexyl) adipate (DEHA)	0003-02	Hexanedioic acid, 1,6-bis(2-ethylhexyl) ester	C_22_H_42_O_4_	103-23-1
Di(2-ethylhexyl) adipate (DEHA)	0004-01	Hexanedioic acid, 1,6-bis(2-ethylhexyl) ester	C_22_H_42_O_4_	103-23-1
Di(2-ethylhexyl) adipate (DEHA)	0004-02	Hexanedioic acid, 1,6-bis(2-ethylhexyl) ester	C_22_H_42_O_4_	103-23-1
Bis(2-ethylhexyl) adipate (DEHA)	0005-02	Hexanedioic acid, 1,6-bis(2-ethylhexyl) ester	C_22_H_42_O_4_	103-23-1
Dichloromethane (methylene chloride) (DCM)	0077-01	-	CH_2_Cl_2_	75-09-2
Dichloromethane (methylene chloride) (DCM)	0078-01	-	CH_2_Cl_2_	75-09-2
Diethylene glycol dibutyl ether (possible substances alternatives)	0091-01	-	C_12_H_26_O_3_	112-73-2
Diisobutyl hexahydrophthalate (DIBE)	0005-01	Diisobutyl 1,2-cyclohexanedicarboxylate	C_16_H_28_O_4_	70969-58-3
Diisobutyl hexahydrophthalate (DIBE)	0006-01	Diisobutyl 1,2-cyclohexanedicarboxylate	C_16_H_28_O_4_	70969-58-3
Dioctyl azelate (DOZ)	0005-02	-	C_25_H_48_O_4_	103-24-2
Dioctylsebacate (Diethylhexylsebacate) (DEHS)	0002-01	Decanedioic acid, 1,10-bis(2-ethylhexyl) ester	C_26_H_50_O_4_	122-62-3
Dioctylsebacate (Diethylhexylsebacate) (DEHS)	0002-02	Decanedioic acid, 1,10-bis(2-ethylhexyl) ester	C_26_H_50_O_4_	122-62-3
Dioctylsebacate (Diethylhexylsebacate) (DEHS)	0003-01	Decanedioic acid, 1,10-bis(2-ethylhexyl) ester	C_26_H_50_O_4_	122-62-3
Dioctylsebacate (Diethylhexylsebacate) (DEHS)	0003-02	Decanedioic acid, 1,10-bis(2-ethylhexyl) ester	C_26_H_50_O_4_	122-62-3
Dioctylsebacate (Diethylhexylsebacate) (DEHS)	0004-01	Decanedioic acid, 1,10-bis(2-ethylhexyl) ester	C_26_H_50_O_4_	122-62-3
Dioctylsebacate (Diethylhexylsebacate) (DEHS)	0004-02	Decanedioic acid, 1,10-bis(2-ethylhexyl) ester	C_26_H_50_O_4_	122-62-3
DPHP Bis(2-Propylheptyl) Phthalate (DPHP)	0002-01	1,2-Benzenedicarboxylic acid, 1,2-bis(2-propylheptyl) ester	C_28_H_46_O_4_	53306-54-0
DPHP Bis(2-Propylheptyl) Phthalate (DPHP)	0002-02	1,2-Benzenedicarboxylic acid, 1,2-bis(2-propylheptyl) ester	C_28_H_46_O_4_	53306-54-0
DPHP Bis(2-Propylheptyl) Phthalate (DPHP)	0003-01	1,2-Benzenedicarboxylic acid, 1,2-bis(2-propylheptyl) ester	C_28_H_46_O_4_	53306-54-0
DPHP Bis(2-Propylheptyl) Phthalate (DPHP)	0003-02	1,2-Benzenedicarboxylic acid, 1,2-bis(2-propylheptyl) ester	C_28_H_46_O_4_	53306-54-0
DPHP Bis(2-Propylheptyl) Phthalate (DPHP)	0004-01	1,2-Benzenedicarboxylic acid, 1,2-bis(2-propylheptyl) ester	C_28_H_46_O_4_	53306-54-0
DPHP Bis(2-Propylheptyl) Phthalate (DPHP)	0002-01	1,2-Benzenedicarboxylic acid, 1,2-bis(2-propylheptyl) ester	C_28_H_46_O_4_	53306-54-0
DPHP Bis(2-Propylheptyl) Phthalate (DPHP)	0004-02	1,2-Benzenedicarboxylic acid, 1,2-bis(2-propylheptyl) ester	C_28_H_46_O_4_	53306-54-0
Ethyl centralite	0005-02	Urea, N,N’-diethyl-N,N’-diphenyl-	C17 H20 N2O	85-98-3
Isodecyl pelargonate (IDP)	0005-02	Nonanoic acid, 8-methylnonyl ester	C_19_H_38_O_2_	109-32-0
Methyl centralite	0005-02	Urea, N,N’-dimethyl-N,N’-diphenyl-	C_15_H_16_N_2_O	611-92-7
Methyl ethyl ketone (MEK)	0080-01	2-Butanone	C_4_H_8_O	78-93-3
Methyl ethyl ketone (MEK)	0081-01	2-Butanone	C_4_H_8_O	78-93-3
Ortho-xylene	0005-01	Benzene, 1,2-dimethyl-	C_8_H_10_	95-47-6
Ortho-Xylene	0006-01	Benzene, 1,2-dimethyl-	C_8_H_10_	95-47-6
Tributyl citrate (TBC)	0005-02	1,2,3-Propanetricarboxylic acid, 2-hydroxy-, 1,2,3-tributyl ester	C_18_H_32_O_7_	77-94-1

**Table 3:** Danish EPA advisory self-classification of hazardous substance by battery models ^§^.

Endpoint	Advisory Classification	Wording of CLP Classification	Battery Models
MUTAGENICITY			
Mutagenicity	Muta. 2	Suspected of causing genetic defects	Bacterial reverse mutation test (Ames test in S. typhimurium *in vitro*)
			Chromosome aberrations in CHO cells (*in vitro*), commercial model from MultiCASE
			Chromosome aberrations in CHL cells (*in vitro*)
			Mutations in thymidine kinase locus in mouse lymphoma cells (*in vitro*)
			Mutations in HGPRT locus in CHO cells (*in vitro*)
			Micronucleus test in mouse erythrocytes (*in vivo*)
			Comet assay in mouse (*in vivo*)
CARCINOGENICITY			
Carcinogenicity	Carc. 2	Suspected of causing cancer	FDA RCA cancer male rat (*in vivo*), commercial models
			FDA RCA cancer female rat (*in vivo*), commercial models
			FDA RCA cancer male mouse (*in vivo*), commercial models
			FDA RCA cancer female mouse (*in vivo*), commercial models
			Bacterial reverse mutation test (Ames test in S. typhimurium *in vitro*)
			Chromosome aberrations in CHO cells (*in vitro*)
			Chromosome aberrations in CHL cells (*in vitro*)
			Mutations in thymidine kinase locus in mouse lymphoma cells (*in vitro*)
			Mutations in HGPRT locus in CHO cells (*in vitro*)
REPRODUCTIVE TOXICITY			
Reproductive toxicity	Repr. 2	Suspected of damaging fertility or the unborn child	Teratogenic potential in Humans
			Dominant lethal mutations in rodents (*in vivo*)

^§^ All data comes from the Danish EPA’s advisory list for self-classification of hazardous substances [52].

**Table 4:** Alternatives in AoA that used QSARs^∔^ in WoE for higher-tier endpoints.

Consultation Numbers	Applicants	Alternatives
0005-01	DEZA, a.s.	Diisobutyl hexahydrophthalate (DIBE)
0005-02	DEZA, a.s.	Methyl centralite
		Ethyl centralite
		Akardite I
		Akardite II
		Akardite III
		Bis(2-ethylhexyl) adipate (DEHA)
		Acetyl tributyl citrate (ATBC)
		Tributyl citrate (TBC)
		Dioctyl azelate (DOZ)
		Isodecyl pelargonate (IDP)
0006-01	Sasol-Huntsman GmbH & Co. KG	Diisobutyl hexahydrophthalate (DIBE)

**Table 5:** Number of criteria met for QSAR predictions used in WoE by CMR/PBT vPvB endpoints.

Consultation Number	Alternative	No. of Criteria Met	Criteria				
			RSS	Assesses reliability, relevance, adequacy, and quantity	“Pools” information	Conflicting results	Assesses overall package
Mutagenicity							
0005-02	Akardite I	1	-	-	x	-	-
0005-02	Akardite III	2	-	-	x	-	x
0006-01	Diisobutyl hexahydrophthalate (DIBE)	2	-	-	x	-	x
0005-02	Dioctyl azelate (DOZ)	2	-	-	x	-	x
0005-02	Ethyl centralite	2	-	-	x	-	x
0005-02	Isodecyl pelargonate (IDP)	2	-	-	x	-	x
0005-02	Methyl centralite	2	-	-	x	-	x
0005-02	Tributyl citrate (TBC)	2	-	-	x	-	x
0005-01	Diisobutyl hexahydrophthalate (DIBE)	3	-	-	x	x	x
0005-02	Akardite II	4	-	x	x	x	x
0005-02	Acetyl tributyl citrate (ATBC)	-	-	-	-	-	-
0005-02	Bis(2-ethylhexyl) adipate (DEHA)	-	-	-	-	-	-
In vitro gene mutation in bacteria (Ames test)							
0005-02	Akardite III	2	-	-	x	-	x
0005-01	Diisobutyl hexahydrophthalate (DIBE)	2	-	-	x	-	x
0006-01	Diisobutyl hexahydrophthalate (DIBE)	2	-	-	x	-	x
0005-02	Ethyl centralite	2	-	-	x	-	x
0005-02	Isodecyl pelargonate (IDP)	2	-	-	x	-	x
0005-02	Methyl centralite	2	-	-	x	-	x
0005-02	Tributyl citrate (TBC)	2	-	-	x	-	x
0005-02	Akardite II	3	-	-	x	x	x
0005-02	Acetyl tributyl citrate (ATBC)	-	-	-	-	-	-
0005-02	Akardite I	-	-	-	-	-	-
0005-02	Bis(2-ethylhexyl) adipate (DEHA)	-	-	-	-	-	-
0005-02	Dioctyl azelate (DOZ)	-	-	-	-	-	-
Carcinogenicity							
0005-02	Akardite I	1	-	-	x	-	-
0005-02	Akardite III	2	-	-	x	-	x
0005-01	Diisobutyl hexahydrophthalate (DIBE)	2	-	-	-	x	x
0006-01	Diisobutyl hexahydrophthalate (DIBE)	2	-	-	-	x	x
0005-02	Dioctyl azelate (DOZ)	2	-	-	x	-	x
0005-02	Ethyl centralite	2	-	-	x	-	x
0005-02	Isodecyl pelargonate (IDP)	2	-	-	x	-	x
0005-02	Methyl centralite	2	-	-	x	-	x
0005-02	Akardite II	3	-	-	-	x	x
0005-02	Tributyl citrate (TBC)	3	-	-	-	x	x
0005-02	Acetyl tributyl citrate (ATBC)	-	-	-	-	-	-
0005-02	Bis(2-ethylhexyl) adipate (DEHA)	-	-	-	-	-	-
Reproductive toxicity							
0005-01	Diisobutyl hexahydrophthalate (DIBE)	1	-	-	-	-	x
0005-02	Methyl centralite	1	-	-	-	-	x
0005-02	Acetyl tributyl citrate (ATBC)	2	-	-	x	-	x
0005-02	Akardite I	2	-	-	x	x	-
0005-02	Akardite III	2	-	-	x	-	x
0006-01	Diisobutyl hexahydrophthalate (DIBE)	2	-	-	-	x	x
0005-02	Ethyl centralite	2	-	-	x	-	x
0005-02	Akardite II	3	-	x	x	-	x
0005-02	Dioctyl azelate (DOZ)	3	-	-	x	x	x
0005-02	Isodecyl pelargonate (IDP)	3	-	-	x	x	x
0005-02	Tributyl citrate (TBC)	3	-	-	x	x	x
0005-02	Bis(2-ethylhexyl) adipate (DEHA)	5	x	x	x	x	x
Ready biodegradability							
0005-02	Akardite I	0	-	-	-	-	-
0005-02	Ethyl centralite	0	-	-	-	-	-
0005-02	Methyl centralite	0	-	-	-	-	-
0005-02	Isodecyl pelargonate (IDP)	1	-	-	-	-	x
0005-02	Acetyl tributyl citrate (ATBC)	-	-	-	-	-	-
0005-02	Akardite II	-	-	-	-	-	-
0005-02	Akardite III	-	-	-	-	-	-
0005-02	Bis(2-ethylhexyl) adipate (DEHA)	-	-				
0005-01	Diisobutyl hexahydrophthalate (DIBE)	-	-	-	-	-	-
0006-01	Diisobutyl hexahydrophthalate (DIBE)	-	-	-	-	-	-
0005-02	Dioctyl azelate (DOZ)	-	-	-	-	-	-
0005-02	Tributyl citrate (TBC)	-	-	-	-	-	-
Bioaccumulation							
0005-02	Akardite I	0	-	-	-	-	-
0005-02	Ethyl centralite	0	-	-	-	-	-
0005-02	Methyl centralite	0	-	-	-	-	-
0005-02	Isodecyl pelargonate (IDP)	1	-	-	-	-	x
0005-02	Dioctyl azelate (DOZ)	2	-	x	-	-	x
0005-02	Acetyl tributyl citrate (ATBC)	-	-	-	-	-	-
0005-02	Akardite II	-	-	-	-	-	-
0005-02	Akardite III	-	-	-	-	-	-
0005-02	Bis(2-ethylhexyl) adipate (DEHA)	-	-	-	-	-	-
0005-01	Diisobutyl hexahydrophthalate (DIBE)	-	-	-	-	-	-
0006-01	Diisobutyl hexahydrophthalate (DIBE)	-	-	-	-	-	-
0005-02	Tributyl citrate (TBC)	-	-	-	-	-	-

Notes: (a) This study only covered QSAR predictions in WoE, thus, hyphenated blank spaces meant that either there were no CMR/PBT vPvB QSAR predictions in WoE to analyze, which were coded as non-applicable, the endpoint data not relevant to WoE, e.g., it evaluates a potential alternative, or a QSAR prediction did not exist to analyze the WoE. (b) Numbers 1–5 correspond to the number of criteria that was met on the checklist, where the total number of criteria was five. (c) 0 indicates a QSAR prediction without a WoE context cited for that endpoint.

**Table 6:** Matching AoA and Danish EPA advisory list for self-classification of hazardous substances ^§^ CAS numbers for CMR endpoints.

CAS	Alternative Substances	C (QSAR) ^∔^	M (QSAR) ^∔∔^	R (QSAR) ^∔∔∔^	CLP Classification
number	(as listed in AoA)				
10025-73-7	Chromium(III) chloride	No	No	No	
102-07-8	Akardite I (1,2-diphenyl urea)	No	No	No	
103-23-1	Bis(2-ethylhexyl) adipate (DEHA)	No	No	Yes	Repr. 2
103-24-2	Dioctyl azelate (DOZ)	No	No	No	
109-32-0	Isodecyl pelargonate (IDP)	No	No	No	
112-73-2	Diethylene glycol dibutyl ether (possible substances alternatives)	No	No	No	
1195978-93-8	Benzene, ethenyl-, polymer with 1,3-butadiene, brominated (brominated co- polymer of styrene and butadiene) (Polymeric Flame Retardant (pFR)	No	No	No	1195978-93-8
122-62-3	Dioctyl sebacate (diethylhexyl sebacate) (DEHS)	No	No	No	122-62-3
13114-72-2	Akardite II (3-methyl-1,1,-diphenyl urea)	No	No	No	13114-72-2
18168-01-9	Akardite III (3-ethyl-1,1,-diphenyl urea)	No	No	No	18168-01-9
29063-28-3	Octanol (mixed isomers) (possible substances alternatives)	No	No	No	29063-28-3
367-23-7	1,2,4-Trifluorobenzene	No	No	No	367-23-7
53306-54-0	Bis(2-propylheptyl) Phthalate (DPHP)	No	No	Yes	Repr. 2
611-92-7	Methyl centralite	No	No	No	
70969-58-3	Diisobutyl hexahydrophthalate (DIBE)	No	No	No	
75-09-2	Dichloromethane (methylene chloride) (DCM)	No	No	No	
77-90-7	Acetyl Tributyl Citrate (ATBC)	No	No	Yes	Repr. 2
77-94-1	Tributyl citrate (TBC)	No	No	Yes	Repr. 2
78-93-3	Methyl ethyl ketone (MEK)	No	No	No	
85-98-3	Ethyl centralite	No	No	No	
95-47-6	Ortho-xylene	No	No	No	

^∔^ C (QSAR) QSAR predictions for carcinogenicity. ^∔∔^ M (QSAR) predictions for mutagenicity. ^∔∔∔^ R (QSAR) predictions for reproductive toxicity. ^§^ All data comes from the Danish EPA’s advisory list for self-classification of hazardous substances. Green boxes indicate matching CAS number in the Danish EPA advisory list.

**Table 7:** Reproductive toxicity identifiers in AoA with matching CAS numbers.

				Reproductive Toxicity Identifiers (AoAs)		
CAS #	Alternative Substance (listed in AoA)	Danish EPA CLP classification	Consultation number(s)	Yes	No	Uncertain
			0002-01	X		
			0002-02	X		
			0003-01	X		
103-23-1	Di(2-ethylhexyl) adipate (DEHA)/Bis(2-ethylhexyl) adipate (DEHA)	Repr. 2	0003-02	X		
			0004-01	X		
			0004-02	X		
			0005-02			X
			0002-01		X	
			0002-02		X	
			0003-01		X	
53306-54-0	Bis(2-Propylheptyl) Phthalate (DPHP)	Repr. 2	0003-02		X	
			0004-01		X	
			0004-02		X	
			0002-01		X	
			0002-02		X	
			0003-01		X	
77-90-7	Acetyl Tributyl Citrate (ATBC)	Repr. 2	0003-02		X	
			0004-01		X	
			0004-02		X	

## Data Availability

Not applicable.

## Supplementary Materials

The following supporting information can be downloaded at: https://www.mdpi.com/article/10.3390/ijerph19074338/s1, Supplementary S1. ITS Comparative Analysis; Supplementary S2. Consultation Numbers; Supplementary S3. Sample WoE Template; Supplementary S4. Study Limitations; Supplementary S5. Sub-Criteria Coding. References [85,86,87,88,89,90] are cited in the supplementary materials.

## Abbreviations

AoA	analysis of alternatives
ATBC	acetyl tributyl citrate
CMR	carcinogenic, mutagenic, reproductive toxic
BCF	bioconcentration factor
BfR	German Federal Institute for Risk Assessment
BIOWIN	Biodegradation Probability Program
CAS	Chemical Abstracts Service
CoRAP	Community Rolling Action Plan
Danish EPA	Danish Environmental Protection Agency
DEHA	bis(2-ethylhexyl) adipate
DIBE	diisobutyl hexahydrophthalate
DPHP	bis(2-propylheptyl) phthalate
DQD	Danish (Q)SAR Database
EC	European Community
ECHA	European Chemicals Agency
EPI Suite^TM^	Estimation Program Interface Suite
EU	European Union
GRADE	Grading of Recommendations Assessment, Development, and Evaluation
INTS	Integrated Non-Testing Strategies
IRIS	Integrated Risk Information System
ITS	integrated systems
IUCLID	International Uniform ChemicaL Information Database
IUPAC	International Union of Pure and Applied Chemistry
LOE	lines of evidence
MCDA	multi-criteria decision analysis-based
MITI	Ministry of International Trade and Industry models
MOA	mechanisms of action
NGO	non-governmental organization
NRC	National Resource Council
OECD	Organisation for Economic Cooperation and Development
PBT/vPvB	persistent, bioaccumulative and toxic or very persistent and very bioaccumulative
QMRF	QSAR Model Reporting Formats
QPRF	(Q)SAR Prediction Reporting Format
QSAR	quantitative structure–activity relationship
REACH	Registration, Evaluation, Authorization, and Restriction of Chemicals
RSS	Robust Study Summaries
SMILES	Simplified Molecular Input Line Entry System
SVHC	Substances of Very High Concern
TBC	tributyl citrate
TCNES	Technical Committee on New and Existing Chemical Substances
TERIS	Teratogen Information System database
U.S. EPA	United States Environmental Protection Agency
WoE	weight of evidence
