# Supplementary material for: Multi-Strategy Assessment of Different Uses of QSAR under REACH Analysis of Alternatives to Advance Information Transparency
Source: Int J Environ Res Public Health. 2022 Apr 4;19(7):4338. doi: 10.3390/ijerph19074338 (PMC8998180; doi:10.3390/ijerph19074338)
Supplement: Supplementary file 1 [file ijerph-19-04338-s001.zip › ijerph-1583701-supplementary.pdf]

Supplementary Materials

Additional information on the comparison of CAS numbers (Supplementary A.1), ITS comparative analysis (Supplementary A.2), consultation numbers (Appendix A.3), a sample WoE template (Supplementary A.4), study limitations (Supplementary A.5), and sub-criteria coding for five main criteria (Supplementary A.6). Appendix A.1. Comparison of CAS Numbers

+ C (QSAR) QSAR predictions for carcinogenicity, ++ M (QSAR) predictions for mutagenicity, +++ R (QSAR) predictions.

Supplementary A.1. ITS Comparative Analysis

To verify information, the name of the alternative substance, as listed in the AoA Table of Contents, was inputted into SciFinder®'s substance identifier search engine, which generated a profile. The CAS number from the profile was then used to verify if the given CAS number in the AoA correctly identified the alternative. If SciFinder® could not find a match based on the alternative's name, the alternative's CAS number was queried and the molecular formula was used to confirm the correct identity of the alternative. If the query did not provide a molecular formula, the EC, IUPAC, or one of the "other" names was used, as given in the AoA, to search for a matching CAS number; however, this was the least reliable method for confirming the correct identity of the alternative. Due to the repeating, non-unique alternatives, each alternative was verified by consultation number.

Supplementary A.2. Consultation Numbers

0005-02 methyl centralite for Genetic toxicity: In vivo—Chromosomal effect, the applicant wrote "equivocal" instead of "inconclusive", and also reported that the results were in AD. However, after querying the predictions myself in the DQD, I saw that results for that endpoint were out of domain. The applicant therefore appeared to incorrectly report the results.

0005-02 ethyl centralite for Genotoxicity in vivo—For sister chromatid exchange in mouse bone marrow cells, it appears that the applicant reported the same QSAR prediction twice. The applicant also wrote "equivocal" in domain, but the report says "positive out of domain (battery)" [1].

0005-02 Akardite II: QSAR prediction for unscheduled DNA repair response based on a mouse bone marrow sister chromatid exchange assay, from the Danish (Q)SAR Database: Danish (Q)SAR Database battery result reported inconclusive out of domain but applicant stated equivocal in domain. All 3 QSARs (Leadscope, Multicase, and SciQSAR) had "Pos out of domain" [1].

0005-02 Akardite III: Applicant reported equivocal instead of inconclusive and said in domain when the DQD report said out of domain. QSAR prediction for Chinese Hamster Ovary (CHO) cell assay for chromosome aberration test, from the Danish (Q)SAR Database.

0005-02 DOZ: QSAR prediction for chromosomal aberration in an in vitro COMET assay in mouse cells, from the DQD: I queried the predictions for DOZ and could not find a COMET assay for mouse cells

in the DQD's in vitro Genotoxicity Endpoints results. QSAR prediction for chromosome aberration in a Chinese Hamster Ovary (CHO) assay, from the DQD reported negative, in domain for Chromosome Aberrations in Chinese Hamster Ovary (CHO) cells.

0005-02 TBC: QSAR prediction for chromosome aberration in Chinese Hamster Ovary (CHO) cells from the DQD: applicant reported equivocal but still in domain. Results from the DQD reported negative in domain.

Supplementary A.3. Sample WoE Template

Table 2: Optional tabular format for summarising weight of evidence assessment

**Question**		***Insert text of question here***
**Assemble the evidence**	Select evidence	*Briefly summarise the methods used to search, select and extract the evidence (see Note 1)*
	Lines of evidence	*List the line(s) of evidence into which the evidence were assembled for assessment and identify any that are missing (see Note 2)*
**Weigh the evidence**	Methods	*Briefly summarise the method(s) used to weigh the pieces and lines of evidence (see Note 3)*
	Results	*Give a reference to the section of the assessment where the results of weighing the pieces and lines of evidence are presented (see Note 4)*
**Integrate the evidence**	Methods	*Briefly summarise the methods used to integrate the pieces and lines of evidence (see Note 5)*
	Results	*State the conclusions of integrating the evidence for this question (see Note 6)*

Italic descriptions are for guidance only and should be deleted once the table is completed.
Notes cited in the table are presented below.

Table A.3: Optional tabular format for summarising weight of evidence assessment of an emerging contaminant

**Question**		***Hazard identification of an emerging contaminant***
**Assemble the evidence**	Select evidence	Nine QSAR models from two *in silico* platforms and a program for read-across were used to estimate mutagenicity potential (as assessed through bacterial reverse mutation test) of the target compound
	Lines of evidence	Except two, all estimates indicated the compound to be non-mutagenic. The exception was the QSAR model CAESAR within VEGA platform that predicted the compound as mutagenic, and the read-across programme ToxRead that showed one out of five similar compounds to be mutagenic
**Weigh the evidence**	Methods	VEGA provides a quantitative measurement of reliability and values higher than 0.8 ADI are considered more reliable. T.E.S.T. applies a filter to eliminate not reliable predictions. The results obtained from these platforms in this case are therefore reliable. ToxRead indicates the alerts associated with the effect and similar compounds. In case of chemicals with the toxicity value conflicting with the rule, the user should check if there are rules present only in the similar compound and not in the target, explaining the conflicting toxicity value. This is useful to evaluate the relevance of the lines of evidence, disregarding those that are not relevant
	Results	T.E.S.T. results consistently indicated non-mutagenicity. The VEGA models called SARpy and KNN showed higher indices for reliability, also predicted non-mutagenicity. The CAESAR and ISS models within the VEGA models showed relatively lower reliability. ToxRead results show that most of the compounds similar to the target compound were not-mutagenic. The only structural rule for mutagenicity found in one similar compound is not present in the target compound, and therefore is not relevant
**Integrate the evidence**	Methods	The *in silico* estimates have been integrated while considering the reliability and relevance of the individual values, together with the consistency of all the predicted values, to make an informed expert judgement about the probability that the target compound is not-mutagenic
	Results	The large majority of the *in silico* values are in concordance for non-mutagenicity of the target compound. One conflicting estimate is less reliable whereas the other is not relevant to the target compound. Considering all the evidence from this *in silico* assessment, it was concluded by informed expert judgement that the target compound is most likely (about 90% probability) to be non-mutagenic

Figure S1. A sample WoE using a structured evidence table [2].

Supplementary A.4. Study Limitations

Unless the description of the QSAR prediction was clearly ad hoc and quantitative values are missing for predictions where there was

already evidence that the QSAR source produces qualitative predictions (for example, consultation 0078-01 where the 1,2,4-trichorobenzene prediction by BIOWIN 3 lacked a qualitative value and only indicated a biodegradation time frame predicted by BIOWIN 3 of "months and longer" for the fluoroisomer compared to "weeks to months" for the chlorinated benzene) [3] I assumed that qualitative and quantitative values reported in AoA by applicants were the results generated by QSAR platforms. However, in select instances, I could not go back and confirm the QSAR sources if they were not identified by model name and version. For example, in AoA consultation number 0005-02, or methyl centralite, the applicant reported "unknown" for irritation: eye irritation. Perhaps the applicant meant "inconclusive" for which I accepted "equivocal" in instances of DQD predictions. Model endpoint information may also have been embedded in an AoA, but this is information typically found in a QMRF. Without having this official document or access to the original QSAR platform, I could not verify if sporadic information in an AoA was what the developer actually described as the model endpoint. In cases where an applicant reported a QSAR prediction twice, for example, consultation number 0005-02 for the alternative ethyl centralite Irritation: Skin irritation/corrosion [4], I accepted it as two separate predictions because I did not have the QPRF to verify the prediction either way.

In addition, all endpoints were based on the curated list of endpoints from Chapter 2. While endpoints outside of this list may have excluded potential QSAR predictions from this analysis, the consistency of this endpoint classification allowed us to subject previous research to a more in-depth analysis without further classification. However, if applicants did not report a specific endpoint, such as in a scoring table, this endpoint did not get coded. For instance, in consultation 0005-02, the applicant did not consider test results to be mutagenic, and subsequently left off mutagenicity from Table 4.31 [4], thereby removing it from our coding.

Furthermore, our approach to data mining and coding may have excluded some aspects of an applicant's WoE from our analysis. When analyzing each AoA, we maintained a narrow research path, and did not track criteria outside of our classifications. For instance, for information to be considered under the final criteria "Assess overall WoE package," the applicant needed to make their scientific arguments within an AoA "Reduction in overall risk." For example, in consultation number 0005-02, scientific arguments were made for the alternative, Akardite I; however, the applicant made these arguments in the comparison of hazards sections. Therefore, any scientific argument written under the "Comparison of Hazards" section would not be coded under the "Assessment of Overall WoE" criteria but to another criteria such as criterion 3, "Pools information". Similarly, for the same consultation number, biodegradability QSAR predictions for the alternative ethyl centralite are not discussed in either the RSS or Reduction of overall risk even though PBT and vPvB is discussed in the WoE for the alternative, ethyl centralite [4]. As we are only considering CMR and vPvB endpoints in AoA that use WoE with QSAR predictions, this information did not get coded. Furthermore, we did not always have the coding to explain when criteria were not met. Although some AoA did not meet our five criteria, in other instances, the criteria simply did not exist in the AoA. For example, in consultation number 0005-02 for the alternative ethyl centralite, we

coded the WoE for the reproductive toxicity endpoint as not providing any conflicting results. However, we assigned this code because all of the information in the AoA for this endpoint was consistent, which would then have been coded separately for "consistence."

If an applicant specified certain endpoints in their approach to WoE, only these endpoints could be considered when judging if the evidence met any of our five WoE criteria. For instance, in consultation 0005-02, the applicant specified Table 4.86 as the basis for "additional insight" into the alternative isodecyl pelargonate (IDP); thus, we could not factor any other endpoint that was not on this list into our completeness review, when considering "adequacy" or the usefulness of the information. More specifically, we could not code either biodegradation or bioaccumulation for "adequacy" because their QSAR predictions were listed on another table [5].

Finally, because the Danish EPA was used to screen for potential CMR substances, our study's results were subject to the factors that went into Danish EPA's ITS QSAR model development, which has been subject to updates since 2001. For example, endocrine disrupting (ED) models, which were not included in the Danish EPA's battery of QSAR models for reproductive toxicity, may be considered an important endpoint in reproductive toxicity for other model developers [6]. Even so, ED models have been used to identify mechanisms for reproductive toxicity [7]. Thus, while other ITS QSAR model developers may have taken a different approach in selecting endpoints for battery QSAR modeling as well as the selection of algorithms to integrate results, results were based wholly on the Danish EPA's decision making that went into their ITS QSAR model development. In addition, our sample of AoA was collected through May 2017; more current AoA may have employed ITS QSAR modeling.

Supplementary *A.5. Sub-Criteria Coding*

Table S2. Sub-criteria coding for the five main weight of evidence criteria.

Weight of Evidence and Five Main Criteria	Sub-criteria
Higher Tier Endpoint (0=No WoE, 1=yes WoE, 2=combination, 3=non-applicable)	0. QSAR but no WoE used;
	1. QSAR and yes WoE used;
	2. Combination of yes WoE and no WoE;
	3. No QSAR predictions made for this specific endpoint or property so WoE analysis is not relevant; or it is unknown if the prediction is from a QSAR.
Criteria[ǂ] (0= no criteria, 1=one criteria, 2=two criteria, 3=3 criteria, 4=4 criteria, 5=all criteria, 6=non-applicable)	0. None of the 6 criteria was addressed in the WoE; this was not a "robust" summary but just a summary;
	1. At least one of the criterion was addressed in the WoE;
	2. At least two of the criteria were addressed in the WoE;
	3. At least three of the criteria were addressed in the WoE;
	4. Greater than or equal to four criteria were addressed in WoE;
	5. All criteria were addressed in WoE;
	6. Endpoint data not relevant to WoE e.g. evaluates potential alternative or info not used in WoE context or no WoE; the six criteria are: 1) Robust study summary, 2) fully documented, 3) objectives, 4) methods, 5) results.

Robust summary (1=objectives, 2=methods, 3=results, 4=conclusions, 5=documentation, 6=non-applicable)	1. Objectives of all test studies in RSS;
	2. Methods of all test studies in RSS;
	3. Results of all test studies in RSS;
	4. Conclusions of a full study report in RSS;
	5. Provides documentation (i.e. copies of the studies);
	6. Non-applicable endpoint data not relevant to WoE e.g. evaluates potential alternative or info not used in WoE context
Assess reliability, relevance, adequacy, quantity (1=reliability, 2=relevance, 3=adequacy, 4=quantity, 5=consistency, 6=severity effects, 7=non-applicable)	1. Reliability: clarity and plausibility of the finding (ECHA 2016 p. 11);
	2. Relevance: data and tests are appropriate for a particular hazard identification (ECHA 2016 p. 11);
	3. Adequacy: usefulness of data for hazard/risk assessment purposes (ECHA 2016 p. 11);
	4. Quantity: number of sources (ECHA 2016 p. 11);
	5. Consistency of results especially within lines of evidence and categories of alternative data e.g. QSAR (ECHA 2016 p. 24);
	6. Severity of the type of effects of concern (ECHA 2016 p. 24);
	7. Non-applicable: endpoint data not relevant to WoE e.g. evaluates potential alternative or info not used in WoE context.
Lines of evidence or structured evidence tables (1=lines of evidence, 2=tables, 3=non-applicable)	1. Lines of evidence (LOE) set of relevant items of information of similar type grouped to assess a hypothesis (Martin et al 2018 p. 076001-5);
	2. Tables displays individual pieces of evidence or categories of evidence for a hypothesis, such as the types shown here, scored with respect to properties or sub-properties and the overall weight (Suter 2017 p. 1041);
	3. Non-applicable; endpoint data not relevant to WoE e.g. evaluates potential alternative or info not used in WoE context.
Conflicting results (1=conflicting results, 2=scoring table, 3=health effect, 4=non-applicable)	1. Addresses conflicting results Note that high quality in vivo (read-across information) and in vitro data would generally carry more weight in the decision than a QSAR or an in-house in vitro method.;
	2. Scoring results: a weighting system e.g. + and - symbols to represent evidence that, respectively, supports, weakens, or has no effect on the credibility of a hypothesis (MUST include the endpoint in question) (Suter 2017 p. 1041);
	3. Health effects from endpoint be inferred from any weighting;
	4. Non-applicable; endpoint data not relevant to WoE e.g. evaluates potential alternative or info not used in WoE context;

Assesses overall package (1= assess, 2= scientifically argued, 3=expert judgment, 4=non-applicable)	1. Composed an assessment of overall hazard of alternative of interest factoring in this endpoint e.g. Conclusions or Reduction of Overall Risk;
	2. Scientifically argued: conclude whether the combined evidence is enough to draw a conclusion about the properties or the potential effects of the substance (ECHA 2016 p. 23);
	3. Expert judgment (considers the reliability, relevance and adequacy, integrating and comparing different pieces of information and assigning a weight to each piece of data) (ECHA 2016 p. 23),
	4. Non-applicable: endpoint data not relevant to WoE e.g. evaluates potential alternative or info not used in WoE context

ᵻ Depending on the number of sub-criteria, not all of the criteria scale was applied.

1. DTU Food (Technical University of Denmark National Food Institute); Danish EPA (Danish Environmental Protection Agency); Nordic Council of Ministers; ECHA (European Chemicals Agency) Danish (Q)SAR Database. http://qsar.food.dtu.dk (March 27, 2022).
2. EFSA Scientific Committee; Hardy Anthony; Benford Diane; Halldorsson Thorhallur; Jeger Michael John; Knutsen Helle Katrine; More Simon; Naegeli Hanspeter; Noteborn Hubert; Ockleford Colin, Guidance on the use of the weight of evidence approach in scientific assessments. *EFSA Journal* **2017,** 15, (8), https://doi.org/10.2903/j.efsa.2017.4971.
3. Dow Italia Srl; Rohm and Haas France S.A.S., Analysis of Alternatives Public Version: Industrial use as a sulphonation swelling agent of polystyrene-divinylbenzene copolymer beads in the production of strong acid cation exchange resins. In 2016.
4. DEZA A.S. *Analysis of Alternatives Non-Confidential Report: Use in propellants: Sub-scenario 1: F-2: Industrial use as a burning rate surface moderant, plasticiser and/or coolant in the formulation of nitrocellulose-based propellant grains. Sub-scenario 2: IW-2: Industrial use of propellant grains in manufacture of ammunition for military and civilian uses, and pyrocartridges for aircraft ejection seat safety systems [excludes propellants intended for manual reloading of ammunition cartridges by civilian users]*; Consultation number 0005-02; n.d.; https://echa.europa.eu/applications-for-authorisation-previous-consultations (27 March, 2022).
5. DEZA A.S. *Analysis of Alternatives Non-Confidential Report: Use as an absorption solvent in a closed system in the manufacture of maleic anhydride (MA)*; Consultation number 0005-01; n.d.; https://echa.europa.eu/applications-for-authorisation-previous-consultations (27 March, 2022).
6. Evans, T. J., Reproductive toxicity and endocrine disruption of potential chemical warfare agents. In *Handbook of Toxicology of Chemical Warfare Agents*, Gupta, R. C., Ed. Academic Press: London, United Kingdom, 2020; pp 641-657.
7. Jensen, G. E.; Niemelä, J. R.; Wedebye, E. B.; Nikolov, N. G., QSAR models for reproductive toxicity and endocrine disruption in regulatory use–a preliminary investigation. *SAR and QSAR in Environmental Research* **2008,** 19, (7-8), 631-641, https://doi.org/10.1080/10629360802550473.
